# Nanoscale bubble domains with polar topologies in bulk ferroelectrics

**DOI:** 10.1038/s41467-021-23863-w

**Published:** 2021-06-15

**Authors:** Jie Yin, Hongxiang Zong, Hong Tao, Xuefei Tao, Haijun Wu, Yang Zhang, Li-Dong Zhao, Xiangdong Ding, Jun Sun, Jianguo Zhu, Jiagang Wu, Stephen J. Pennycook

**Affiliations:** 1 Department of Materials Science, Sichuan University, Chengdu, China; 2 State Key Laboratory for Mechanical Behavior of Materials, Xi’an Jiaotong University, Xi’an, China; 3 Department of Materials Science and Engineering, National University of Singapore, Singapore City, Singapore; 4 Instrumental Analysis Center of Xi’an Jiaotong University, Xi’an Jiaotong University, Xi’an, China; 5 School of Materials Science and Engineering, Beihang University, Beijing, China

**Keywords:** Ferroelectrics and multiferroics, Electronic properties and materials

## Abstract

Multitudinous topological configurations spawn oases of many physical properties and phenomena in condensed-matter physics. Nano-sized ferroelectric bubble domains with various polar topologies (e.g., vortices, skyrmions) achieved in ferroelectric films present great potential for valuable physical properties. However, experimentally manipulating bubble domains has remained elusive especially in the bulk form. Here, in any bulk material, we achieve self-confined bubble domains with multiple polar topologies in bulk Bi_0.5_Na_0.5_TiO_3_ ferroelectrics, especially skyrmions, as validated by direct Z-contrast imaging. This phenomenon is driven by the interplay of bulk, elastic and electrostatic energies of coexisting modulated phases with strong and weak spontaneous polarizations. We demonstrate reversable and tip-voltage magnitude/time-dependent donut-like domain morphology evolution towards continuously and reversibly modulated high-density nonvolatile ferroelectric memories.

## Abstract (web summary)

Experimentally manipulating bubble domains remains elusive especially in the bulk form of ferroelectrics. Here, the authors achieve self-confined bubble domains with multiple polar topologies in bulk Bi0.5Na0.5TiO3 ferroelectrics, demonstrating reversible and donut-like domain morphology evolution.

## Introduction

For ferroic materials, spatial variations in the orientation of order parameters (strain, polarization, and magnetization) are accommodated through the formation of discrete domains^1–6^. Separated by domain walls, normally, different orientations of ordering vectors are expected in adjacent different domains, while they are constant within the same domain^1–6^. However, this conventional notion of ferroic domain microstructure is not the only way in which ordering vectors can be arranged spatially^1–10^. Nano-sized ferroelectric bubble domains, presenting polar topologies (e.g., vortices, skyrmions), were recently achieved in thin-film superlattices of (PbTiO_3_)_*n*_/(SrTiO_3_)_*n*_^2–5^. However, such domain structures have not so far been achieved in a bulk material. If it were possible to stabilize these structures, we could achieve multi-conformational domain structures with multitudinous physical properties, e.g., chirality, ultrahigh memory density (exceeding tens of Tb in^−2^), low-power consumption, and colossal electromechanical activity^2–5,11,12^, in economical bulk materials for practical applications. In ferromagnetic materials, the Dzyaloshinskii−Moriya interaction can be used as the driving force for stabilizing and eventually manipulating these bubble domains, but is not available in ferroelectrics. To trigger the continuous rotation of electrical dipoles in ferroelectric materials, alternative driving forces must be found, which have been forecasted by several researchers and are becoming a major endeavor in recent years^13,14^. Thin films allow better control over the ferroelectricity and depolarization field, and by varying the epitaxial constraint between the ferroelectric and paraelectric layers, polar bubble domains with various polar topologies have been observed in high-quality multilayer thin films^3–5^. Nevertheless, no such effective epitaxial constraint is applicable to bulk materials, which narrows the experimental options for triggering and further utilizing these topological configurations in bulk ferroelectrics.

Theoretical and experimental findings suggest that kinetic constraint possibly exists in systems that exhibit two or more distinct modulated phases, and therefore should also apply to bulk materials^15^. Relaxor ferroelectrics, with decoupled dipoles (of multiple symmetries) naturally coexisting in local regions^16^, may just satisfy the requirement of this kinetic constraint. In their free energy expansion, the high-order gradient terms could provide a negative wall energy, which could drive modulations of polarization vectors as well as domain configurations^15^. Regulated by this constraint, labyrinthine and stripe domain structures can be achieved in thin-film or bulk relaxor ferroelectrics, but not yet for bubble domains^15,17–19^. Determined by a critical balance among elastic, electrostatic, and gradient energy terms, bubble domains might be stable only within a very narrow range of boundary conditions. It leads to the long-standing challenge of capturing such special domain structure in relaxor ferroelectrics^2–5,19^, especially for bulk ferroelectrics that lack the epitaxial constraint of multilayer thin films.

Bi_0.5_Na_0.5_TiO_3_-based relaxor ferroelectrics possess concomitantly modulated short-range-ordered crystal symmetries (mainly *R3c* and *P4bm*), based on which, we have manipulated the domain conformations through modifying the local structural heterogeneity. Labyrinthine, stripe, bubble, and even polar-skyrmion domains are demonstrated using a combination of Z-contrast imaging and phase-field modeling. We experimentally validate the possibility of triggering the bubble domains in bulk ferroelectrics, achieving multiple polar topologies, especially polar skyrmions. The total free energy of bulk ferroelectrics is mainly affected by the competition of bulk, elastic, and electrostatic energy terms of coexisting modulated phases, which determines the observed bubble domains, especially for vortices or skyrmions. Not only do we demonstrate the possibility of triggering the bubble domains in bulk ferroelectrics, but also our results demonstrate that Bi_0.5_Na_0.5_TiO_3_-based relaxor ferroelectrics represent a material family exhibiting polar skyrmions. It opens up a direction to understand the puzzles on topologies (e.g., skyrmions) and design functionalities previously inaccessible in bulk materials. Here, we propose one possibility, manipulating the reversible and tip-voltage magnitude/time-dependent donut-like domain morphologies, which holds potential for designing high-density nonvolatile ferroelectric memories that can be continuously and reversibly modulated. In addition to the prerequisite requirement for applications, reversibility, the ability for continuous modulation, holds promise for creating a series of memory states, superior to the conventional “0” and “1” states.

## Results

**Fig. 1:**
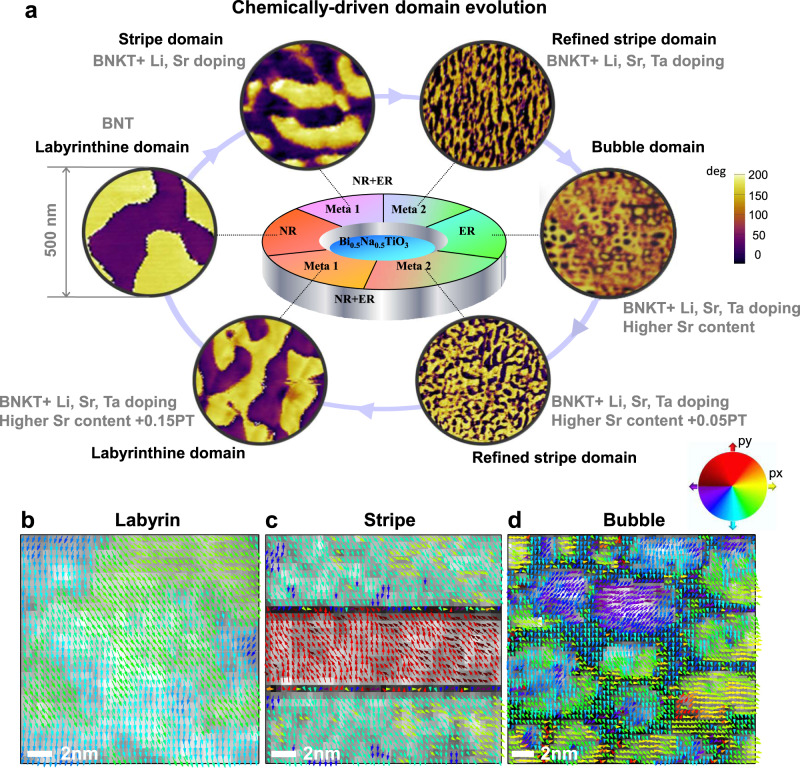
Observation of bubble domains in bulk ferroelectrics. **a** Chemically driven reversible domain evolution in bulk Bi_0.5_Na_0.5_TiO_3_-based ferroelectrics. **b**–**d** Results derived from atomically resolved STEM ABF (contrast-inverted) images (Supplementary Fig. 7a−c) of BNT-labyrinthine, BNT-stripe, and BNT-bubble samples along [110]_pc_, with the *δ*_Ti-O_ displacement vector maps overlaid on their corresponding polarization intensity; the displacement vectors are indicated as colored arrows according to their rotation angles, and the polarization intensity is indicated by bright−dark contrast.

Using piezo-force microscopy (PFM), the composition-induced domain evolution is demonstrated by the induced phase change (Fig. 1a, detailed topological and amplitude information can be seen in Supplementary Fig. 2), which ties in closely with the transition from the nonergodic relaxor (NR) state to the ergodic relaxor (ER) state^20,21^. For easy description, we abbreviate the chemical formulas for different compositions, and the details are provided in the “Methods” section. By doping appropriate elements, the domain pattern can be regulated from large-size labyrinthine domains [BNT, NR state], to refined stripe domains [BNKT, metastable state (Meta) 1, consisting of NR and ER states where NR plays the dominant role], then to ultrafine stripe domains (BNKLSTT, Meta 2, consisting of NR and ER states where ER plays the dominant role), and finally to bubble domains (BNKLSTT-higher Sr, ER state). Moreover, these bubble domains (more discussions about the bubble domains can be seen in Supplementary Figs. 3−5) can be transformed back to the large-sized labyrinthine domains through the opposite path, as PbTiO_3_ is further introduced into the bubble composition. Notably, during this domain transition process, we also observe a reversible trend in electrical properties accordingly (Supplementary Fig. 6).

Having the obvious morphology evolution of labyrinthine, ultrafine stripe, and bubble domains from a macroscopic level, their local polarization states turn out to be critical to reveal the structural details of the chemically driven domain evolution. For ferroelectric materials, their local structures are determined by local polarizations. For Bi_0.5_Na_0.5_TiO_3_-based materials, the displacement of the center Ti^4+^ cation with respect to the corner Bi^3+^/Na^+^ cations (*δ*_Ti-Bi/Na_) or the center of its nearest O^2-^ neighbors (*δ*_Ti-O_) can be used to represent the local polarization state (Supplementary Fig. 1c). To directly see the local atom displacement, aberration-corrected scanning transmission electron microscopy (STEM) was employed due to its ultrahigh resolution and *Z*-contrast feature for direct atom identification (Supplementary Fig. 1c). Furthermore, the large probe-forming aperture available on our system allows a depth of field of around 5 nm, assisting us to image a thin section through the 3D domain structure.

For a better view, the polarization vectors are overlaid on their corresponding intensity (|*P* | = $\sqrt{{({\mathrm{d}}x)}^{2}+{({\mathrm{d}}y)}^{2}}$), which clearly shows the local structure transition from large-sized labyrinthine (Fig. 1b) to refined stripe (Fig. 1c) and then to bubble (Fig. 1d) domains. In analogy to the domain wall of traditional ferroelectric domains, a domain wall width of about 1−2 unit cells can still be observed for the stripe domains (Fig. 1c), while the domain wall is curved and broadened for bubble domains (Fig. 1d).

Polarization vectors of bubble domain boundaries present a continuous rotation, in complete contrast to those of the well-defined traditional ferroelectric domains^2^. Consistent with the observations by PFM, low-magnitude TEM bright-field images of representative BNT-bubble samples present arrays of self-confined circular domains (inset of Supplementary Fig. 8). Statistical analysis of domain width distribution (Supplementary Fig. 8) obtained from PFM and low-magnitude TEM images indicates that the average size of the bubble domains with clear boundaries is around 25 nm (after adding approximate width of tiny round-shaped domains without clear boundaries, the average size is around 16 nm). The combined PFM and TEM images demonstrate that the chemically driven domain morphology evolution and the observed self-confined bubble-like feature clearly differentiate Bi_0.5_Na_0.5_TiO_3_-based materials from their peers.

**Fig. 2:**
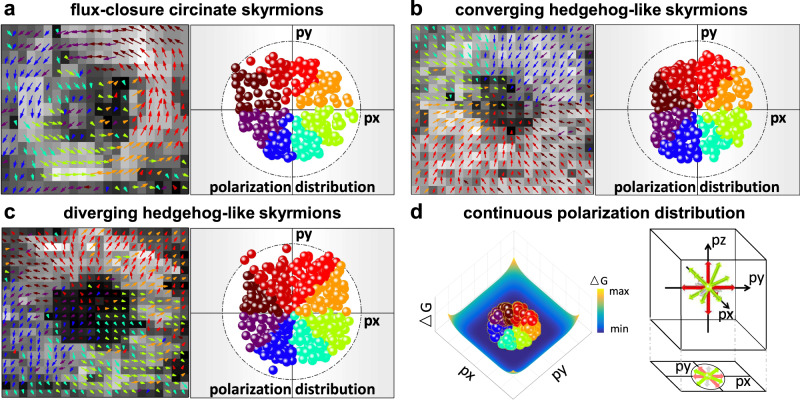
Polymorphous configurations of bubble domains. Polymorphous conformational topologies of polarization vectors observed in bubble domains, with three representative polar skyrmions shown, including **a** continuous electric dipole rotation in a circinate flux-closure Bloch-like form, **b** converging hedgehog-like form, and **c** diverging hedgehog-like form. The polarization vectors are filled with different colors according to their angles (left), which exhibit a continuous distribution (right). The grid background shows the corresponding magnitude of each vector. **d** Phenomenological illustration of the continuous polarization distribution. Due to the highly flattened free-energy landscape, the coexistence of continuously distributed polarizations is observed.

The polarization vectors of bubble domains show several representative conformational topologies, e.g., arranging along one particular direction and rotating continuously among different directions. Most significantly, we find continuous electric dipole rotation showing flux-closure Bloch-like-skyrmion topologies (Fig. 2a and Supplementary Fig. 9a, b), which have never been reported in any bulk material. Moreover, in some areas, the polarization vectors converge from the edge to the center (Fig. 2b and Supplementary Fig. 9c), exhibiting the form of a converging hedgehog-like skyrmion structure^4^. Polarization vectors also propagate away from a disclination point (Fig. 2c and Supplementary Fig. 9d), showing the form of a diverging hedgehog-like skyrmion structure^5^. These observations of bubble domains point to the formation of polar-skyrmion structures with both a Bloch-like component, where the polarization vectors rotate smoothly in a flux-closure form (Fig. 2a and Supplementary Fig. 9a, b), and hedgehog-like Néel components, where the polarization vectors rotate smoothly from the center to the edge or vice versa (Fig. 2b, c and Supplementary Fig. 9c, d). It is noted that the nanodomains with various polar topologies are much smaller than domains observed in both PFM (Fig. 1a) and low-magnification TEM (Supplementary Fig. 8). The Wenny domains with fuzzy boundaries in the larger scale are possibly consisted with finer nanodomains whose polarizations have certain preference direction on average. Such hierarchical domain pattern would be highly sensitive to the applied electric field, which actually involves more complex and synergistic movements among the nanodomains with multiple polar topologies.

**Fig. 3:**
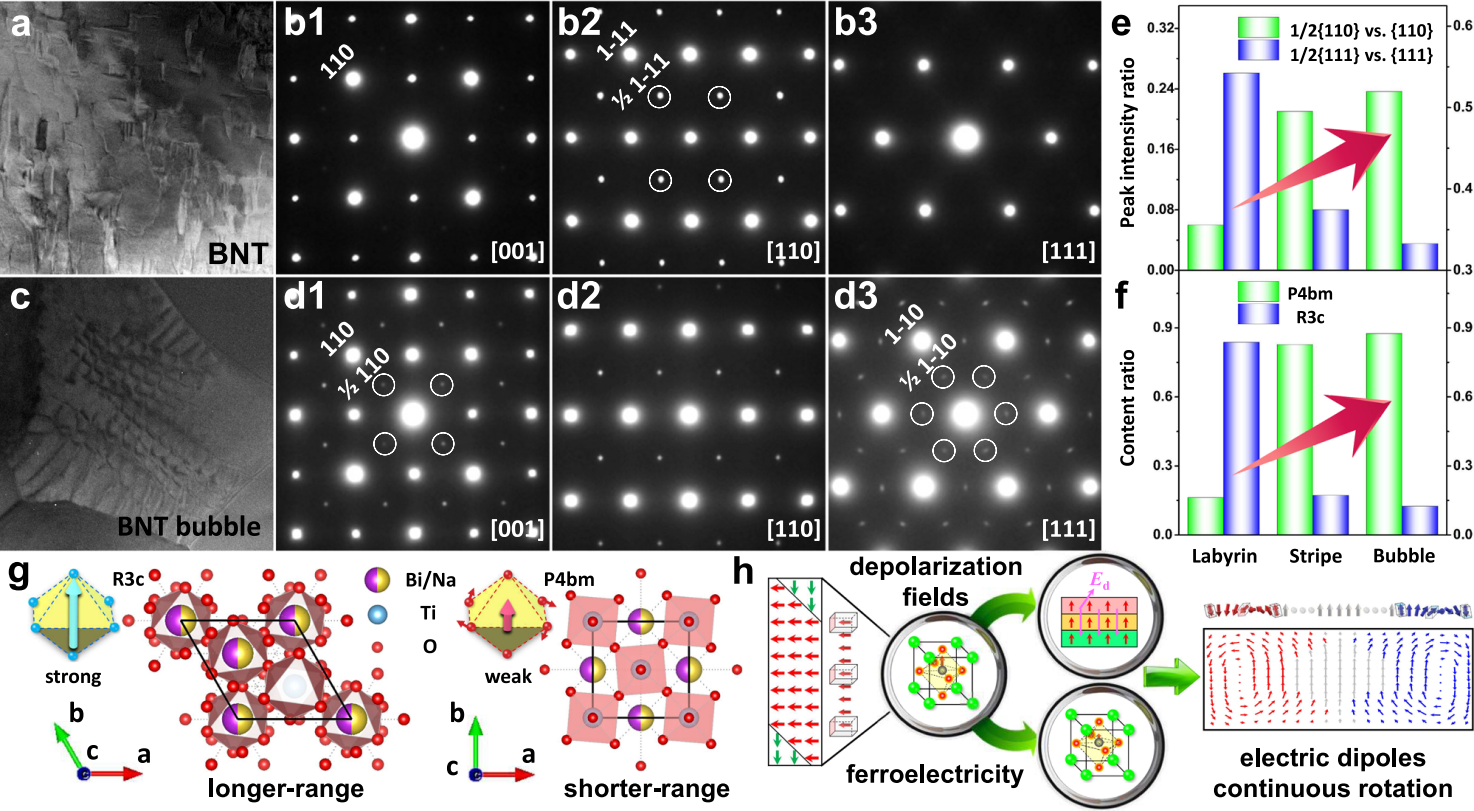
Structural essence accompanied with the evolutionary domains. TEM bright-field images of the representative **a** BNT-labyrinthine and **c** BNT-bubble samples. **b1−b3**, **d1−d3** Electron diffraction patterns of BNT-labyrinthine and BNT-bubble samples, respectively, along [001]_pc_, [110]_pc_, and [111]_pc_ zone axes. **e** Peak intensity ratios of {110} vs. ½{110} and ½{111} vs. {111}reflections. **f** Relative ratios of *P4bm* and *R3c* content for BNT-labyrinthine, BNT-stripe, and BNT-bubble samples. **g** Structural models of R (*R3c)* and T (*P4bm*) phases viewed along *c* axis. **h** Driving forces utilized to achieve bubble domains in bulk ferroelectrics, and the expected continuous rotation of electrical dipoles in space.

The observed polarization behaviors raise fundamental questions on their physical origin. According to phenomenological theory, relaxor ferroelectrics with coexisting symmetries have higher-order gradient terms in their free-energy expansion^8,15,22–25^, compared to those with a single symmetry, which could further flatten the free-energy landscape and naturally give rise to polarization modulation (Fig. 2d). Slightly deviated from the parent perovskite cubic symmetry, short-range ordered crystal symmetries (rhombohedral *R3c* and tetragonal *P4bm*, Fig. 3g) of Bi_0.5_Na_0.5_TiO_3_-based materials can be revealed by the presence of superlattice ½{*ooo*}-type and ½{*ooe*}-type (*o* and *e* stand for odd and even Miller indices) reflections, respectively^22,23^. Selected-area electron diffraction (SAED) patterns along three basic zone axes ([001]_pc_, [110]_pc_, [111]_pc_) reveal the increased intensity of ½{*ooe*}-type reflections with a decreased intensity of ½{*ooe*}-type reflections from BNT-labyrinthine to BNT-stripe and then to BNT-bubble samples, as shown in Fig. 3b1−b3, d1−d3. More precisely, peak intensity ratios of ½{110} over {110} and ½{111} over {111} are obtained (Fig. 3e) through intensity profiles whose width covers both main spots and the reflection spots. The enhanced or lowered intensity of ½{*ooe*}-type or ½{*ooo*}-type diffraction spots indicates the increasing ratio of the shorter-range-correlated *P4bm* symmetry, which is consistent with the high-resolution X-ray diffraction Rietveld refinement analysis (Fig. 3f and Supplementary Fig. 10). Due to the antiparallel displacement between A-site and B-site ions with respect to the oxygen octahedron, *P4bm* symmetry exhibits ferrielectric features and has a weak spontaneous polarization^24,25^, while *R3c* symmetry shows ferroelectric features with a strong polarization^23–25^. To minimize the polarization discontinuity, the interfacial energies (including electrostatic, elasti, and gradient energies) favor the polarization modulation between the coexisting symmetries. Therefore, *P4bm* symmetry plays a crucial role in restraining the domain boundary, as the depolarization field does, while *R3c* determines the main body of the bubble domains. The increasing ratio of *P4bm* is expected to segment the large-sized domains. If *R3c*/*P4bm* symmetries coexist in a critical ratio range, bubble domains or even polar skyrmions can be realized in Bi_0.5_Na_0.5_TiO_3_-based bulk ferroelectrics. As revealed by the macro and local structural analysis, the domain morphology evolution is tightly correlated with the ferroelectric−ferrielectric symmetry transition.

Below, we combine phase-field modeling and theoretical rationalization to reveal the physical origin of the above-mentioned observations, including (1) composition-induced domain evolution, (2) microscopic features of the bubble domains, and (3) the skyrmion-like topological conformations.

**Fig. 4:**
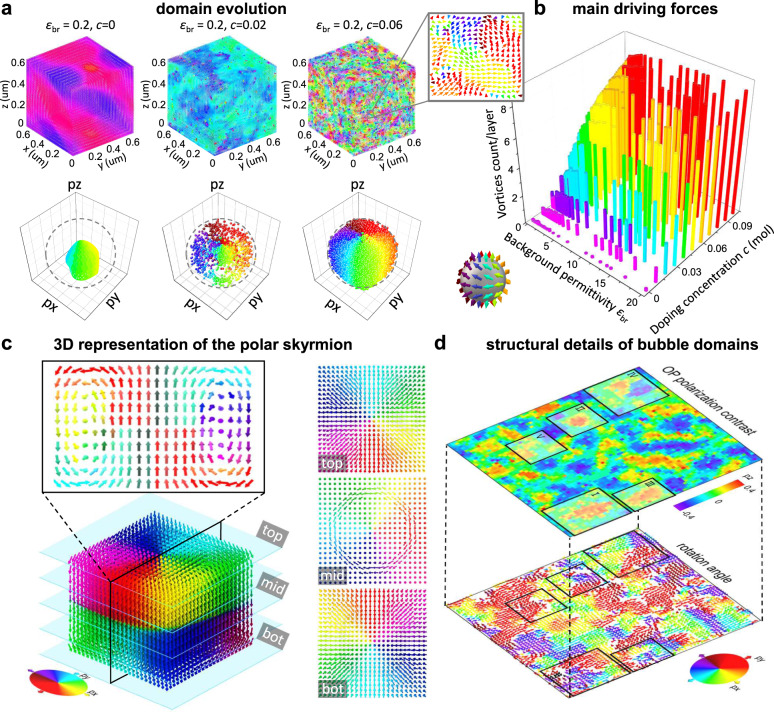
Origin and structural details of bubble domains. **a** The upper part shows the domain evolution with increasing doping concentration, and the lower part shows the corresponding polarization distribution. The expanded region (upper-right corner) shows the local polarization distribution derived from the bubble-like domain structure. **b** Main driving forces to form the bubble-like domain structure. **c** 3D representation of a polar skyrmion selected from the bubble domains. **d** Theoretical reconstruction of the bubble domains. Regions I−V are selected regions, which reflect the multiple topological configurations in the bubble domains. More domain evolution and driving forces and structural details can be seen in Supplementary Figs. 11−17.

With phase-field modeling, we reproduce the composition-regulated evolution of domain patterns, from the large-sized labyrinthine-like domains to the refined stripe-like domains and finally to the distinctive bubble-like domains with polar topologies (upper part of Fig. 4a and Supplementary Fig. 11). Consistent with the continuously distributed two-dimensional (2D) polarizations of bubble domains (Fig. 2a−c), the three-dimensional (3D) polarization of a highly doped system also exhibits a similar continuous distribution (bottom part of Fig. 4a). The expanded region derived from a slice of the bubble-like domains shows a clear vortex structure, as shown in the upper-right corner of Fig. 4a.

The origin of the bubble domains is explored based on time-dependent Ginzburg−Landau simulations. Generally, the total free energy of bulk ferroelectrics can be decomposed into bulk, gradient, elastic, and electrostatic energies^26–28^, and the competition of these energy terms determines the most stable domain configuration. Briefly, we consider a prototypical ferroelectric material with multiple symmetries described by a polarization field and then introduce the doping-induced defects as built-in random fields of various magnitudes. By considering and comparing several factors (background dielectric permittivity, doping concentration, doping intensity, doping range, and anisotropy factor) that determine the energy terms, it is found that the doping concentration *c* greatly influences the number of vortices and the background dielectric permittivity *ε*_br_ (Fig. 4b and Supplementary Figs. 12−15) is the second influence factor. Due to the strong strain–polarization coupling in ferroelectrics, the local stress field induced by doping plays an important role in adjusting the ratio of IP (in-plane) and OP (out-of-plane) polarizations (Supplementary Fig. 25). The electrostatic interactions favor the IP dipole orientation, which rationalizes the fact that a larger *ε*_br_ for Bi_0.5_Na_0.5_TiO_3_-based solid solutions is helpful to regulate the domain conformations. These combined factors substantially influence the formation of polar bubble domains and even vortices or skyrmions.

The main driving forces to regulate the domain conformations lie in the interplay of the bulk, elastic, and electrostatic energies of coexisting modulated phases^13,14,26–28^. Based on the phase-field modeling results for bubble domains, the typical case, polar skyrmions, is presented here. Figure 4c displays a 3D representation showing the polar skyrmion-like topological conformations. The distributions of IP and OP polarizations are clearly revealed from this 3D perspective. The up and down OP polarizations are smoothly connected by IP polarizations. Consistent with the experimental observations (Fig. 2b, c), polar regions with diverging and converging polarizations (Néel form) are observed at the top and bottom of the skyrmion, respectively. In the middle of the skyrmion, IP polarizations rotate continuously (Bloch form), smoothly bridging the up and down OP polarizations. The combined experimental and theoretical results demonstrate the existence of bubble domains and even polar skyrmions in bulk Bi_0.5_Na_0.5_TiO_3_-based solid solutions. Such peculiar 3D topological structure differentiates from the ordinary 2D skyrmions observed in magnetic systems^29^. Moreover, the polar skyrmion structure seen here is a particular case of the various bubble domains observed in the present bulk system^30,31^. In addition to the 2D flux-closure vortices (regions I−IV in Fig. 4d) and 3D flux-closure skyrmions (Fig. 4c), the polarization vectors also rotate in non-flux-closure form (region V in Fig. 4d and Supplementary Fig. 16). Collectively, these topological conformations constitute the bubble domains with multiple polar topologies observed in bulk Bi_0.5_Na_0.5_TiO_3_-based solid solutions, as demonstrated by both experimental (Figs. 1–3) and theoretical (Fig. 4) observations.

Bubble domains, often seen as the initial state of the ferroelectric domain-switching process, are kinetically unstable and difficult to be captured^2–5^. The bubble domains are stable only within a narrow range of boundary conditions determined by a balance among elastic, electrostatic, and gradient energy terms. In this case, the bubble domain state would be easily affected by external forces (chemical pressure, mechanical pressure, voltage, etc.)^2–5,8^. Due to the *E*-field-induced polarization rotation from the IP to OP directions, such metastable and short-range correlated bubble-like topological patterns are expected to experience an *E*-field-induced conformational transition, leading to the enhanced piezoelectric responses during the voltage-loading process^3,8^. To test the local piezoelectric response and conformational transition during the polarization rotation process, switching spectroscopic PFM (SSPFM) hysteresis behavior was investigated on the bubble domains (Supplementary Fig. 18g). In the first hysteresis cycle, the amplitude signal increases steeply from 0 to −20 V, which signifies that the bubble domains are undergoing the polarization rotation process and could give large piezoelectric responses. Phase images inset in Supplementary Fig. 18g were derived from local regions before and after PFM hysteresis loop measurement, which demonstrate the transformation from bubble to cylindrical domain states. It is worth mentioning that the bubble domains surrounding the formed cylindrical domain, far away from the voltage tip, can coalesce into the large-sized anti phase domains, see the reverse contrast of the phase image (Supplementary Fig. 18h, i). Such stable alternating current (A.C.) voltage-induced antiphase domain morphology makes our bubble domains achieved in bulks distinctive in ferroelectrics^3^. It would be easier to obtain multifarious domain morphologies by manipulating relative sizes of anti phase domains in bulk Bi_0.5_Na_0.5_TiO_3_-based ferroelectrics, without injecting a large amount of charges through long-time high-voltage or under humid conditions^28,29^.

**Fig. 5:**
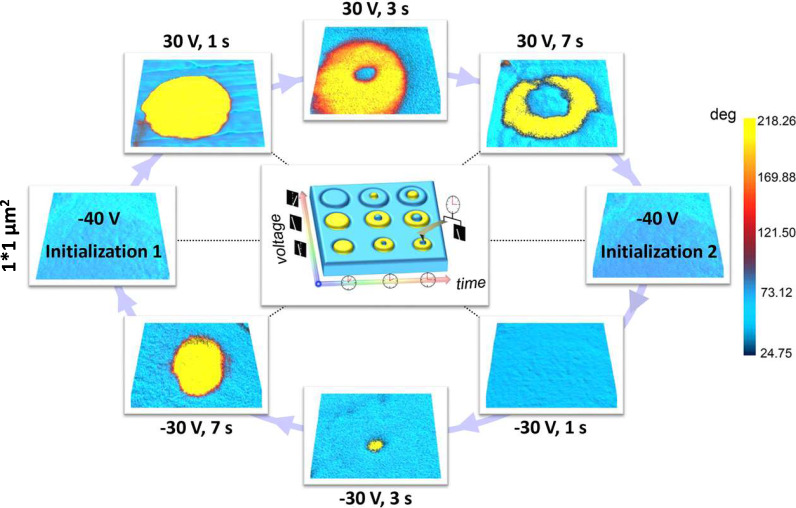
Potential applications toward high-density ferroelectric memories. The voltage-induced domain evolution was performed by PFM. A preprocessing −40-V voltage was first applied to a 1*1-μm^2^ bubble region for the initialization, then a tip voltage of (±30 V, 1−7 s) was applied to one point to trigger donut-like topological domains with opposite polarizations. The whole process is reversible. Detailed tip-voltage-manipulated evolution of domain morphologies can be seen in Supplementary Figs. 17−19. Tests are carried out under dry atmosphere condition (humidity = 0%).

In addition to the A.C. voltage utilized in the SSPFM test, a direct current (D.C.) voltage was then applied to the bubble regions to manipulate the domain morphologies. A preprocessing −40-V voltage was first scanned over a 1 μm*1 μm bubble region for the initial poling. Then, a unidirectional voltage is applied to one point. Controlling the duration and magnitude of the applied voltage induces peculiar donut-like antiphase domains around this point (Fig. 5 and Supplementary Figs. 18−22). The ferrielectric *P4bm* symmetry weakens polarizations along the same direction, and its antiparallel polarizations are present to minimize the total free energy, which is notably different from that of the BNT-stripe (Supplementary Fig. 23) and BNT-labyrinthine (Supplementary Fig. 24) samples. The magnitude of the tip voltage determines the width of the outer ring, while the application time determines the width of the inner part. Moreover, such tip voltage-induced phase and amplitude contrast of the donut-like domains can be maintained after 72 h (Supplementary figure 22c), indicating good stability of such peculiar voltage-induced self-conformation from the initial bubble domains. More importantly, these tip voltage-manipulated domain morphologies are rewritable. By scanning a large opposite voltage over the same region, the domain morphologies could well return to the initial state.

The observed multifarious peculiar domain morphologies present at least three important features to note, including: (1) continuously varying ferroelectric domain morphologies can be easily manipulated by the duration and magnitude of the applied tip voltage, (2) the donut-like anti-phase domains are spatially stable and experimentally recoverable, and (3) two groups of the donut-like domain morphologies induced by opposite tip voltages exhibit mirrored patterns. These results, therefore, demonstrate the concept of a continuously and reversibly modulated high-density nonvolatile ferroelectric memories, whose operation is linked to the above reversible tip-voltage magnitude/time-dependent domain morphology evolution.

In summary, we report the direct observation of polar bubble domains including polar skyrmions in bulk Bi_0.5_Na_0.5_TiO_3_-based ferroelectrics. This is the first observation of the elusive polar skyrmions, composed of a series of diversely oriented Néel- and Bloch-like conformations, in any bulk ferroic material. The coexisting *R3c*/*P4bm* symmetries significantly affect the interfacial energies, and *R3c* determines the main body of the bubble domains, while *P4bm* restrains the domain boundary. Driven by the interplay of bulk, elastic, and electrostatic energies of coexisting modulated phases, such peculiar topological structures clearly differentiate from the ordinary 2D skyrmions observed in magnetic systems, as well as the 3D skyrmions observed in multilayer thin films. By controlling the duration and magnitude of the applied tip voltage, these polar topologies exhibit donut-like domain morphologies that open up the concept for potential applications: beyond reversibility, necessary for the conventional “0” and “1” states, the possibility for non-conventional and high-density nonvolatile ferroelectric memories is based on sequential modulation through a series of memory states.

## Supplementary information

Supplementary Information

## Data Availability

The data corresponding to this study are available from the first author and corresponding authors on request.
