# Author Correction: Nanoscale bubble domains with polar topologies in bulk ferroelectrics

**DOI:** 10.1038/s41467-021-24356-6

**Published:** 2021-06-24

**Authors:** Jie Yin, Hongxiang Zong, Hong Tao, Xuefei Tao, Haijun Wu, Yang Zhang, Li-Dong Zhao, Xiangdong Ding, Jun Sun, Jianguo Zhu, Jiagang Wu, Stephen J. Pennycook

**Affiliations:** 1 Department of Materials Science, Sichuan University, Chengdu, China; 2 State Key Laboratory for Mechanical Behavior of Materials, Xi’an Jiaotong University, Xi’an, China; 3 Department of Materials Science and Engineering, National University of Singapore, Singapore City, Singapore; 4 Instrumental Analysis Center of Xi’an Jiaotong University, Xi’an Jiaotong University, Xi’an, China; 5 School of Materials Science and Engineering, Beihang University, Beijing, China

**Keywords:** Ferroelectrics and multiferroics, Electronic properties and materials

Correction to: *Nature Communications* 10.1038/s41467-021-23863-w; published online 15 June 2021

The original version of this Article misspelled the following number in the Acknowledgements: ‘52032007’, which incorrectly read ‘52032007, 52032007‘. This has now been corrected in both the PDF and HTML versions of the Article.

