# Supplementary material for: Nanoscale bubble domains with polar topologies in bulk ferroelectrics
Source: Nat Commun. 2021 Jun 15;12:3632. doi: 10.1038/s41467-021-23863-w (PMC8206216; doi:10.1038/s41467-021-23863-w)
Supplement: Supplementary file 1 — Supplementary Information [file 41467_2021_23863_MOESM1_ESM.pdf]

Supplementary information for

Nanoscale bubble domains with polar topologies in bulk ferroelectrics

Jie Yin[1,#], Hongxiang Zong[2,#], Hong Tao[1], Xuefei Tao[2], Haijun Wu[2,3,*], Yang Zhang[4], Li-Dong Zhao[5], Xiangdong Ding[2], Jun Sun[2], Jianguo Zhu[1], Jiagang Wu[1,*] and Stephen J. Pennycook[3,*]

[1]Department of Materials Science, Sichuan University, Chengdu 610064, China.
[2]State Key Laboratory for Mechanical Behavior of Materials, Xi'an Jiaotong University, Xi'an 710049, China.
[3]Department of Materials Science and Engineering, National University of Singapore, Singapore 117575, Singapore.
[4]Instrumental Analysis Center of Xi'an Jiaotong University, Xi'an Jiaotong University, Xi'an 710049, China.
[5]School of Materials Science and Engineering, Beihang University, Beijing 100191, China. [#]These authors contributed equally to this work.
*Corresponding author. Email: wuhaijunnavy@xjtu.edu.cn (H. W.); msewujg@scu.edu.cn (J. W.); stevepennycook@gmail.com (S. J. P)

Methods

Sample Fabrication

Raw materials including Bi_2O_3 (4N), Na_2CO_3 (4N), K_2CO_3 (4N), Li_2CO_3 (5N), $SrCO_3$ (5N), PbO (5.5N) TiO_2 (4N), Ta_2O_5 (5N) were utilized to fabricate samples with BNT-labyrinthine, BNT-stripe, BNT-refined stripe, BNT-bubble, inversed BNT-refined stripe and inversed BNT-labyrinthine domain structures. All of them are provided by Alfa Aesar (China) Chemicals Co., Ltd. Chemical formulas are given as:

BNT-labyrinthine: $Bi_{0.50}Na_{0.50}TiO_3$;

BNT-stripe: $[Bi_{0.50}(Na_{0.72}K_{0.18}Li_{0.10})_{0.50}]_{0.96}Sr_{0.04}TiO_3$;

BNT-refined stripe: $[Bi_{0.50}(Na_{0.72}K_{0.18}Li_{0.10})_{0.50}]_{0.96}Sr_{0.04}Ti_{0.985}Ta_{0.015}O_3$;

BNT-bubble: $[Bi_{0.50}(Na_{0.72}K_{0.18}Li_{0.10})_{0.50}]_{0.90}Sr_{0.10}Ti_{0.985}Ta_{0.015}O_3$;

Inversed BNT-refined stripe: $[Bi_{0.50}(Na_{0.72}K_{0.18}Li_{0.10})_{0.50}]_{0.85}Sr_{0.10}Pb_{0.05}Ti_{0.985}Ta_{0.015}O_3$;

Inversed BNT-labyrinthine: $[Bi_{0.50}(Na_{0.72}K_{0.18}Li_{0.10})_{0.50}]_{0.75}Sr_{0.10}Pb_{0.15}Ti_{0.985}Ta_{0.015}O_3$.

First, raw materials were weighed according to the corresponding chemical formula, and then they were ball milled for 24 h with both Y-stabilized ZrO_2 balls and ethyl alcohol as the media. Next, these dried powders were calcined at 900 °C for 6 h. Mixed with a binder of 5 *wt* % polyvinyl alcohol (PVA), the calcined powders were pressed into pellets (with diameter of about 10 mm and thickness of about 1.0 mm) by uniaxial pressing at 10 MPa, followed by binder burn-out at 550 °C and cold isostatic pressing at 300 MPa. After the removal of PVA, the white pellets were buried in their calcined powders and sintered at 1130 °C for 2 h in air atmosphere and cooled down to room temperature with cooling rate of 10 °C/min. The relative density of the ceramics was measured by the Archimedes method, and all ceramics possess high relative densities (>95%, Extended Data Table 1). To characterize electrical properties, silver paste was pasted on both sides of the samples, and then annealed at 600 °C for 10 min.

Electrical Property Measurements

To measure the piezoelectricity, the samples were subjected to a poling treatment by applying a D.C. (direct current) electric field of 5 kV/mm in a silicon oil bath. Quasi-static d_{33} values were characterized by the Berlincourt method (piezo-meter, ZJ-3A, China, Extended Data Fig. 1a). k_p values were determined by the impedance method (the IEEE Standard) using an impedance analyser (HP4294). The frequency-dependent dielectric signals (ε_r and tan δ) (100 Hz–500 kHz) were characterized by a *LCR* analyzer (Tonghui 2816A, China and HP 4980, Agilent, U.S.A.). The curves of dielectric constant and dielectric loss versus temperature (ε_r–T and tan δ-T) (25–550 °C) were characterized by a programmable furnace with the *LCR* analyzer. The *in-situ* E-field dependence of polarization and bipolar strain (P-E and S-E) hysteresis loops of the samples were measured by a ferroelectric tester (aixACCT TF Analyzer 2000, Germany, Extended Data Fig. 1b) at a frequency of 1 Hz. The impedance spectrum was measured using an impedance analyser (HP4294). Local piezoelectric response was characterized by Piezo-force Microscopy, as shown below.

Structural Characterization

Piezo-force Microscopy (PFM).

For PFM observation, the sintered samples were ground down to about 400 μm and subsequently polished using polycrystalline diamond paste with abrasive particles of 9 μm, 3 μm, 1 μm and 0.3 μm (DP-Paste P by Struers A/S, Ballerup, Denmark) for 30 min each. Ultra-fine polishing (<0.03 μm) was then performed on these pre-polished samples (Presi mecatech 234). The domain morphology was characterized using an atomic force microscope (AFM, MFP-3D, Asylum Research, USA) with PFM functions for local poling experiment and switching spectroscopy. A Pt- and Ir-coated cantilever (NanoWorld EFM, force constant: 2.8 N/m, resonance frequency: 75 kHz) was utilized to do the PFM measurement, using a drive voltage of 2 V. The statistical data (width of each domain) was derived using ImageJ (v1.8.0).

The switching spectroscopy PFM (SSPFM) was used to characterize the local piezoelectric response, with the alternating triangular waves shown in Extended Data Fig. 21b. Before and after the SSPFM test, PFM images were obtained with 2 V as the scanning voltage on the bubble region. The local poling treatment was carried out on the polished surface by the PFM tip. To have a more intuitive image, by using litho-PFM mode, a large poling voltage was applied on the selected area to induce the larger-sized domains first. Then, gradient tip voltages were loaded on selected points (see test waves and illustrations in Extended Data Fig. 21c, 21d). Finally, PFM images were obtained with 2 V as the scanning voltage on the selected area. PFM tests are carried out under dry atmosphere condition (humidity=0%).

(Scanning) Transmission Electron Microscopy (TEM and STEM).

The (S)TEM specimens were prepared by slicing, polishing and then ion milling with liquid N_2 cooling (Fischione M1051 TEM Mill). Aberration-corrected STEM investigation was conducted with a JEOL ARM200F equipped with cold field emission gun and ASCOR probe corrector.

Relationship between atomic displacements and local polarization

For ferroelectric $Bi_{0.5}Na_{0.5}TiO_3$, the spontaneous polarization (P_S) arises from the relative displacements between the centers of negative (O^{2-}) and positive (Bi^{3+}/Na^+ and Ti^{4+}) ions. The displacement of the center Ti^{4+} cation with respect to the corner Bi^{3+}/Na^+ cation ($\delta_{Ti\text{-}Ba/Na}$) is accompanied by the relative displacement of the oxygen octahedron ($\delta_{O\text{-}Ba}$)[33,34]. Therefore, the relative displacement of the center Ti^{4+} cation with respect to the center of the oxygen octahedron ($\delta_{Ti\text{-}O}$) or the center of the Bi^{3+}/Na^+ corners ($\delta_{Ti\text{-}Ba/Na}$) could determine the local polarization (Extended Data Fig. 1c). In the present work, we employ $\delta_{Ti\text{-}O}$ obtained from STEM ABF images. Then the local P_S can be roughly estimated using a linear relation to $\delta_{Ti\text{-}O}$: $Ps = k\delta_{Ti\text{-}O}$, where k is a material-dependent constant[35-39].

To determine the displacement (polarization) vectors, the atomic positions of the atomically resolved STEM images are located accurately by fitting them as 2D Gaussian peaks. The $\delta_{Ti\text{-}O}$ is calculated as a vector between each Ti^{4+} and the center of mass of its O^{2-} neighbors. The directions of $\boldsymbol{P}_S$ vectors can thus be estimated by the $\delta_{Ti\text{-}O}$ vectors pointing from net negative to positive charges. The visualization of the two-dimensional polar displacement vectors was carried out using Matlab.

High-resolution X-Ray diffraction (XRD).

Sintered samples were ground into the powders with sizes of <10 μm, and then

these powders were annealed at 600 °C for 30 min. High-resolution XRD was carried out with CuK_α radiation in the θ–2θ scan mode (Bruker D8 Advanced XRD, Bruker AXS Inc., Madison, WI, Cu K_α) on the corresponding powders.

Theoretical Calculations and Modelling

Phase-field Modelling.

Phase-field simulations of $Bi_{0.5}Na_{0.5}TiO_3$-based solid solutions were used to investigate the skyrmion-like bubble domain structure as well as the condition of its formation. In the present work, the domain structures are described by the spatial distribution of spontaneous polarization P (P_1, P_2, P_3), and the total free energy of the system F can be written as:

$$F = \int dV(f_{bulk} + f_{elas} + f_{grad} + f_{elec} + f_{RF})$$

where f_{bulk} is the bulk free energy density, f_{elas} is the elastic energy density, f_{grad} is the gradient energy density, f_{elec} is the electrostatic energy density and f_{RF} is the contribution of the random field due to chemical disorder, respectively.

The bulk free-energy density is expressed for zero strain as a six-order polynomial expansion, that is

$$f_{\mathrm{bulk}} = \alpha_1(P_1^2 + P_2^2 + P_3^2) + \alpha_{11}(P_1^4 + P_2^4 + P_3^4) + \alpha_{12}(P_1^2P_2^2 + P_2^2P_3^2 + P_1^2P_3^2) + \alpha_{111}(P_1^6 + P_2^6 + P_3^6) + \alpha_{112}[P_1^4(P_2^2 + P_3^2) + P_2^4(P_1^2 + P_3^2) + P_3^4(P_1^2 + P_2^2)] + \alpha_{123}P_1^2P_2^2P_3^2$$

where α_i, α_{ij}, α_{ijk} are the Landau parameters that can be obtained from experiment. The elastic energy density can be written as $f_{\mathrm{elas}} = \frac{1}{2}C_{ijkl}(\varepsilon_{ij}-\varepsilon_{ij}^0)(\varepsilon_{kl}-\varepsilon_{kl}^0)$, where C_{ijkl} is the elastic stiffness tensor, ε_{ij} is the total strain and ε_{ij}^0 is the spontaneous strain during the phase transformation. The spontaneous strain is related to the polarization by the electrostrictive coefficients $\varepsilon_{ij}^0 = Q_{ijkl}P_kP_l$, where Q_{ijkl} is the electrostrictive coefficient. The anisotropy factor $A = C_{44}/C_{11}$. The gradient energy density can be obtained by $f_{\mathrm{grad}} = \frac{1}{2}G_{ijkl}\, P_{i,j}\, P_{k,l}$, where G_{ijkl} is the gradient coefficient.

The electrostatic energy can be expressed as $f_{\mathrm{elec}} = \frac{1}{2}(\boldsymbol{E}\cdot\boldsymbol{P})$, where $\boldsymbol{E}$ is the total electric field, which can be evaluated from the electrostatic potential φ as $\boldsymbol{E} = -\nabla\varphi$. In the model the space is assumed to be charge free, therefore the equation: $\nabla\cdot(-\varepsilon_0\, \varepsilon_{br}\nabla\varphi+\boldsymbol{P}) = 0$ can be used to solve the electric field, where ε_0 and ε_{br} are vacuum and the background relative dielectric constants, respectively.

The random field component is expressed as $f_{RF} = \boldsymbol{E}_{\mathrm{random}}\cdot\boldsymbol{P}$. The random electric field $\boldsymbol{E}_{\mathrm{random}}$ can also be related to the negative gradient of a potential, $\varphi_{\mathrm{random}}$. This potential is, however, excluded from the electric potential φ defined here. The random electric field is assumed to be static and independent from field variables. Here, we assume that the local random electric field caused by individual doping site $\boldsymbol{E}^i_{random}(\vec{r})$ is not restricted to a single mesh point, but influences its surroundings[40]. Accordingly, we assume a spatial Gaussian distribution of the random electric field

$E^{i}_{random}(\vec{r}) = \frac{E_0}{\sigma\sqrt{2\pi}} e^{-\frac{\vec{r}^2}{2\sigma^2}}$, where E_0 is the strength or potency of the doping effect in the host system, and σ determines the range or neighborhood this doping site can influence.

The evolution of polarization is governed by the time-dependent Ginzburg-Landau equation:

$$\frac{\delta P_i(\boldsymbol{r},t)}{\delta t} = -\Gamma \frac{\delta F}{\delta P_i(\boldsymbol{r},t)} \quad (i = 1,2,3)$$

where $\boldsymbol{r}$ is the spatial vector and Γ is the kinetic coefficient. Here, a 64 × 64 × 64 grid mesh of points with periodic boundary condition has been employed for the simulation. The Landau parameters utilized in this work were taken from ref 41, and the elastic coefficients are listed in Extended Data Table 2. Other parameters (E_0, σ, ε_{br}) are continuously tuned to figure out the possible factors that give rise to the presence of bubble domain structure.

Topological density.

The topological density of a 3D skyrmion is calculated according to the following equation:

$$\boldsymbol{q} = \frac{1}{4\pi} P\cdot\left(\frac{\partial P}{\partial x} \times \frac{\partial P}{\partial y}\right)$$

where P denotes the normalized local dipole moment. The skyrmion number, $N_{sk} = \iint \boldsymbol{q}\, dxdy$ was obtained from the local dipoles by computing the normalized polarization field defined along each slice of 64 × 64 × 1 elemental perovskite units in the (001) plane of our supercell[42]. We obtain the vortex number per-layer by $N_{vortex} = \iint |\boldsymbol{q}|\, dxdy$.

Supplementary Text

Discussions about the bubble domains imaged by PFM

The PFM method is based on the detection results of local electromechanical vibration for a ferroelectric/piezoelectric sample. Ferroelectric domains are visualized by monitoring the first harmonics of the deflection signal. Its amplitude, R, is proportional to the local longitudinal piezoelectric coefficient, and the phase ϕ reflects the polarization direction ($\phi \sim 0°$ if the probing E-field is parallel to the local polarization and $\phi \sim 180°$ if the E-field is anti-parallel). Thus, PFM allows for full reconstruction of the domain morphology and local polarization distribution[43].

According to the domain configurations, the bubble-like domains (Extended Data Fig. 2h and 2l) are classified into three types, including the hollow-circle-shaped domains (type 1), the solid-round-shaped domains (type 2) and the tiny-round-shaped domains (type 3) without clear boundaries, as the representative expanded regions shown in Extended Data Fig. 3a-3c. The statistical analysis of domain width distribution for different domain configurations is provided in Extended Data Fig. 3d. The hollow-circle-shaped (10-49 nm) and solid-round-shaped (17-46 nm) configurations exhibit the wider width distribution than the tiny-round-shaped (< 15 nm) one. These nano-sized bubble domains with complex phase contrast surround each

other, which are different from the micron-sized cylindrical domains observed in bulk ferroelectrics[53,54] and the bubble domains emerged from the ferroelectric matrix observed in thin films[45]. As indicated by the STEM polarization analysis, the polarization vectors of bubble domains rotate continuously from one direction to the other (Extended Data Fig. 7c-7e) everywhere, which is obviously different from normal ferroelectric domains (including the cylindrical domains)[45, 53, 54]. Moreover, the local dipoles with opposite directions are also found to be connected through a mixed Bloch-like/Néel-like manner (Extended Data Fig. 9). Based on these observations, continuously rotated dipoles were revealed in the further phase-field modelling to explain the experimental phenomena and to reconstruct the detailed local structures. Extended Data Fig. 3e-3h schematically show the phase-field modelling results. The vortex-like domain structures exhibit different sizes and occurred at several locations in the sample. Due to the short-range-correlated ferroelectric order of the BNT-bubble sample, the dipoles within a large-sized vortex tend to form the ellipsoid-like domain structures rather than the cylindrical domains, and the dipoles within a small-sized vortex tend to form the spheroid-like domain structures. Those rotated dipoles without a closed flux tend to form the irregular structures. This model can explain the observations reported in this work, indicating the interesting and abundant polar topologies in these bubble-like structures.

Discussions about the Reversible Electric Properties

Accompanied with the reversible domain evolution, the corresponding dielectric (Extended Data Fig. 6a and 6h), impedance (Extended Data Fig. 6c, 6i), ferroelectric (Extended Data Fig. 6d), electro-strain (Extended Data Fig. 6e) and piezoelectric (Extended Data Fig. 6g) responses are also reversible, indicating the close correlation between the domain morphology and their physical properties[44].

Discussions about the A.C. and D.C. voltage-induced domain transition

It is reasonable to understand the A.C. (alternating current) voltage-induced domain transition from the bubble state to the cylinder state (insets of Extended Data Fig. 18g), as reported in multilayer thin films[45]. However, the classical theories of relaxor ferroelectrics[46-50] do not provide any explanation for the following behaviors: (1) loading the unidirectional D.C. voltage in one point will induce a donut-like domain conformation with anti-parallel polarization directions (Fig. 5 in the main text and Extended Data Fig. 18), and (2) the bubble-like domains near the A.C. voltage-loading point spontaneously transfer into large-sized domains, with anti-parallel polarization direction to the voltage-induced cylindrical domains (Extended Data Fig. 18h).

These abnormal behaviors may not originate from the relaxor ferroelectricity, but from the large strain energy of bulk $Bi_{0.5}Na_{0.5}TiO_3$-based solid solutions[51]. To minimize the strain energy, voltage-induced domain patterns with anti-parallel polarization directions are expected to be observed.

Domain evolution evidenced by both experimental and theoretical observations

Consistent with the experimental observations (Extended Data Fig. 7, 9), phase

field modelling shows a clear conformational domain transition from the large-sized labyrinthine domains (Extended Data Fig. 11a, 11d) to the refined-sized stripe domains (Extended Data Fig. 11b, 11e) and finally to the bubble-like domains (Extended Data Fig. 11c, 11f). Accompanied with the ultra-small domain sizes, more and more rotation angles can be observed from these polarization vectors. Flux-closure Bloch-skyrmion-like topologies can be seen in the self-confined polar bubble domains, and the diverging and converging polarization vectors of these polar structures resemble the spin configurations of Néel skyrmions in ferromagnets[52]. Results from the phase-field modelling predict the possibility of bubble domains and even the skyrmion-like polar structures.

Based on the ferroelectric material with multi-symmetries, among several factors that determine the energy terms of the total free energy (Extended Data Fig. 12, 13, 14, 15), we found that the main driving forces of the domain evolution lies in the doping concentration and the background dielectric permittivity ε_{br}. The former indicates that the chemical pressure is effective to regulate the domain morphology, and the latter indicates that the intrinsic properties should be also considered when we selected the material system. To decrease the bulk energy term, multi-symmetries need coexist in the ferroelectric material, giving rise to local polarization modulation. Polarization vectors tend to smoothly rotate to accommodate the competition of bulk, elastic, electrostatic and gradient energy terms. Vortex and skyrmion structures are special cases of these topological domain conformations.

Different kinds of topological conformations for bubble domains

The theoretical modelling predicts that the polarization vectors have both flux-closure and non-flux-closure forms (Extended Data Fig. 11c), which is also validated in the experimental observation (Extended Data Fig. 7, 9). Except for the flux-closure rotation sharing the rotation angle of 360°, non-flux-closure rotations sharing different rotation angles (90°, 180°, for example) are observed in these bubble domains. It is worth noting that the polarization vectors rotate smoothly, which differentiates the bubble-like domains observed in $Bi_{0.5}Na_{0.5}TiO_3$-based solid solutions from the normal ferroelectric domains.

To have a better understanding about the structural details of the bubble domains, we reconstruct the bubble domains derived from the phase-field modelling results from a 3D view (Extended Data Fig. 16). Isolated by the IP polarizations (green color contrast), OP polarizations (red and blue color contrast) exhibit the bubble-like conformations with cold and warm color contrast, demonstrating the existence of the polar bubble domains in a clearer way.

Reversibility of the voltage-induced evolutions for bubble domains

As shown in Figure 5, the domain evolution is reversible. A similar domain pattern can re-appear in almost the same region, as Extended Data Fig. 26 shown. Under external electric field, nanoscale bubble domains would transform to larger-scale columnar domains as shown in Figure 5. The whole process is reversible and repeatable in the same region. During the “loading-unloading-loading” process, similar doughnut-

like domain patterns are observed repeatably, which demonstrates that the local features are reversible. Furthermore, the mobility and recoverability of skyrmion-like polar structures has been confirmed by our phase field simulations, as shown in Extended Data Fig. 27-29. Here, we take several skyrmion-like polar structures (red box in Extended Data Fig. 27-29) for example, and we can see that the positions of these structures change a bit with the external field. Without exception, the skyrmion-like polar structures can move back their initial positions after unloading, indicating a recoverable nature of the skyrmion-like polar structures. From this aspect, it agrees well with our PFM experiment (Extended Data Fig. 26).

Density and size of the bubble domains

Extended Data Fig. 30 shows the size of vortex structures in BNT system with three different doping concentration, which can reflect the size of bubble domains with polar topologies. We can find in Extended Data Fig. 30a that size of vortex structures is becoming more uniform as we increase the doping defect concentration. More importantly, the mean size of bubble domains decreases with the increasing doping concentrations, as Extended Data Fig. 30b shown.

These additional discussions demonstrate that these bubble-like conformations are ferroelectric bubble domains with different kinds of topological conformations.

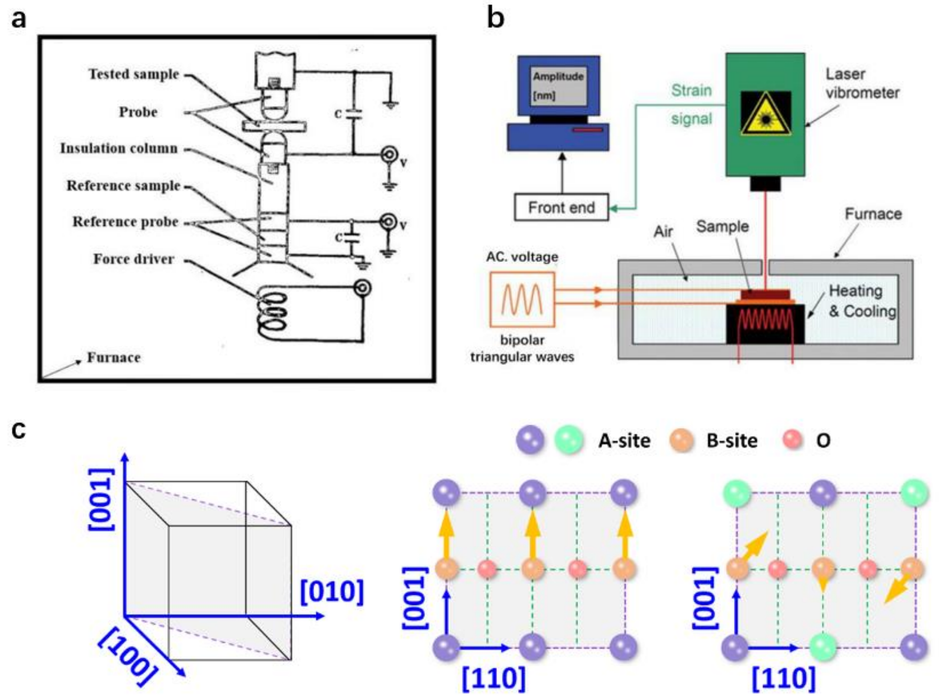

Extended Data Fig. 1 | Instruments utilized to detect the piezoelectric, ferroelectric responses and local polarization distribution. a, Principles of d_{33} meter: The Berlincourt method was utilized to obtain the quasi-static piezoelectric coefficient d_{33} based on the direct piezoelectric effect. **b**, Principles of ferroelectric and electro-strain tester: The laser method was utilized to detect the ferroelectric polarization and electro-strain, and the test waves are provided in Extended Data Fig. 6f. **c**, Schematic illustration showing how to determine the local polarization state, where rotation angle and polarization intensity are two key parameters for polarization vectors.

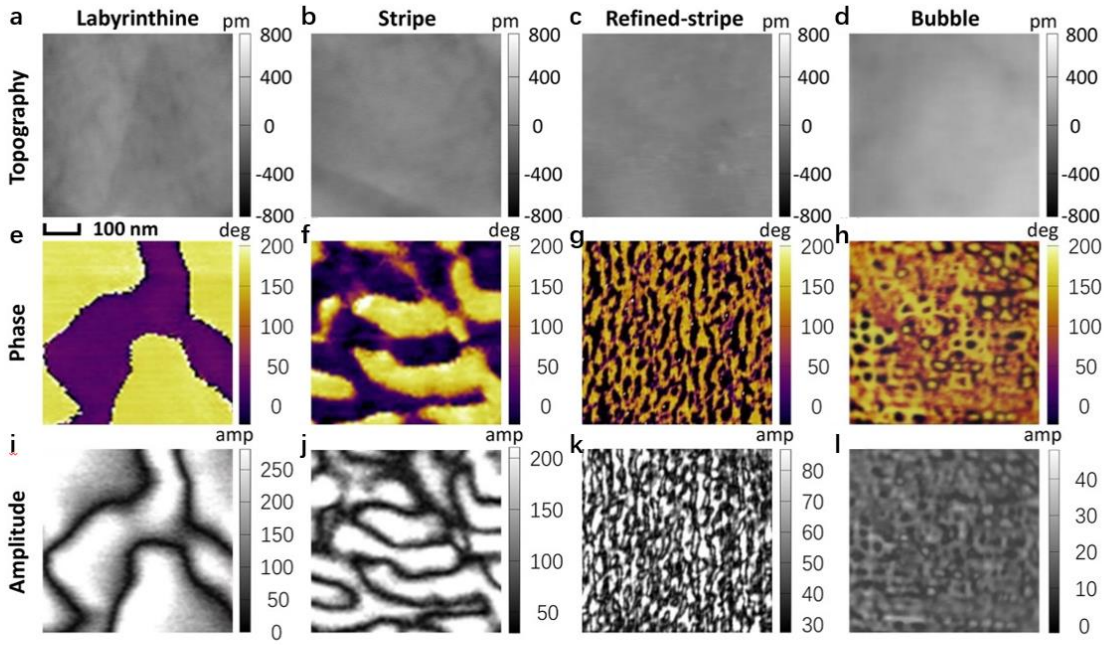

Extended Data Fig. 2 | Detailed PFM images. a-d, Topography, **e-h,** phase and **i-l,** amplitude images for the samples with labyrinthine, stripe, refined-stripe and bubble domain structures.

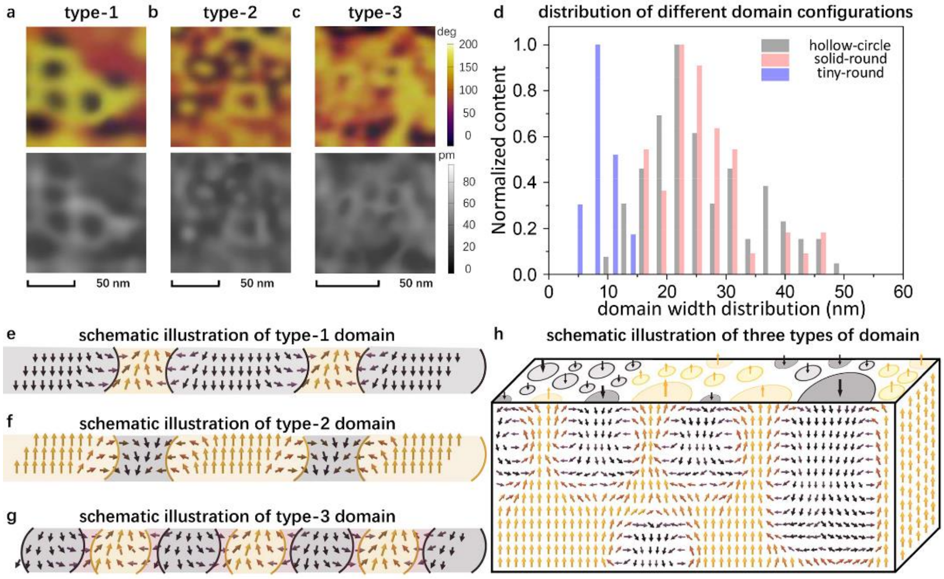

Extended Data Fig. 3 | Classification and explanation of the bubble domains. (a-c) Three different types of domain configurations, including a) hollow-circle-shaped domain, b) solid-round-shaped domain and c) tiny-round-shaped domains. (d) Separated statistics of domain width distribution of the three types of domain configurations. (e-g) Schematic illustration of three types of domain configurations extracted from the PFM, STEM and phase-field modelling results.

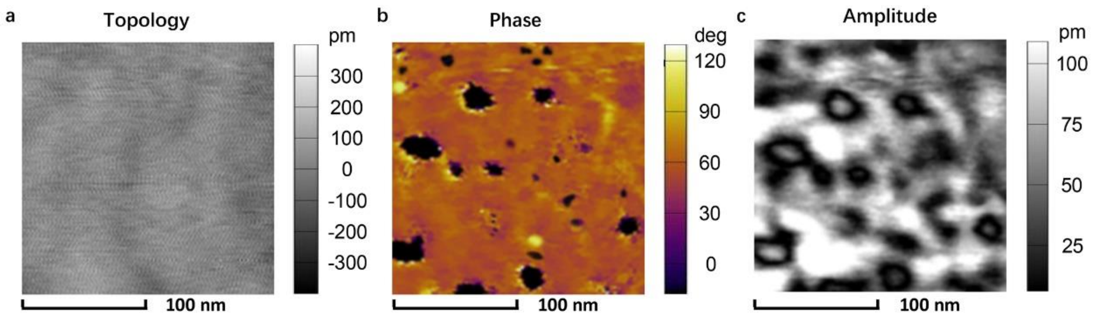

Extended Data Fig. 4 | Supplementary high-resolution PFM images of the bubble domains. (a-c) PFM images reflecting the a) topology, b) phase, and c) amplitude of the selected region with bubble domains.

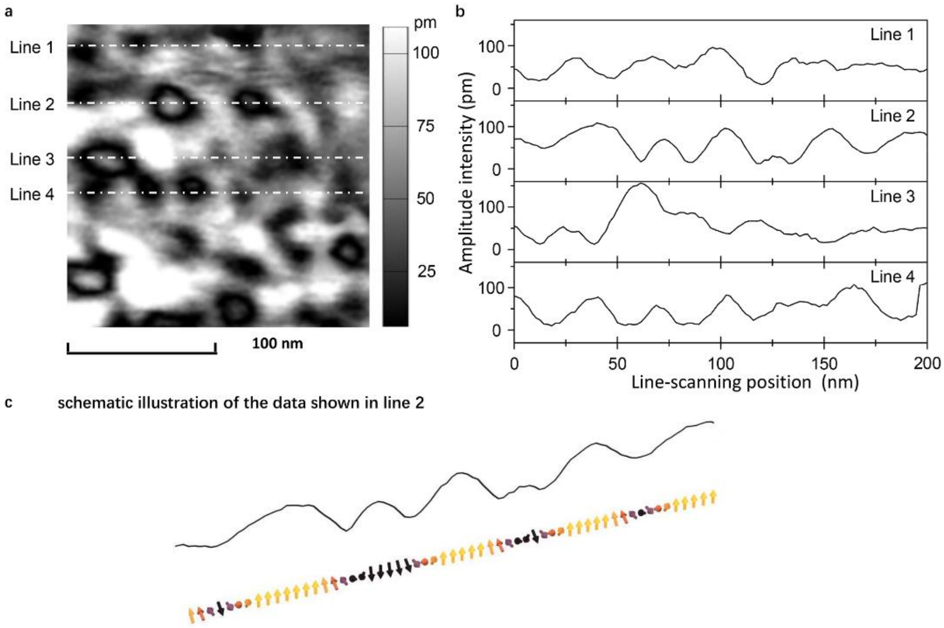

Extended Data Fig. 5 | Line-scanning analysis based on the PFM images. (a) Line position to do the line-scanning, (b) detailed line-scanning data and (c) schematic illustration about the line-scanning data.

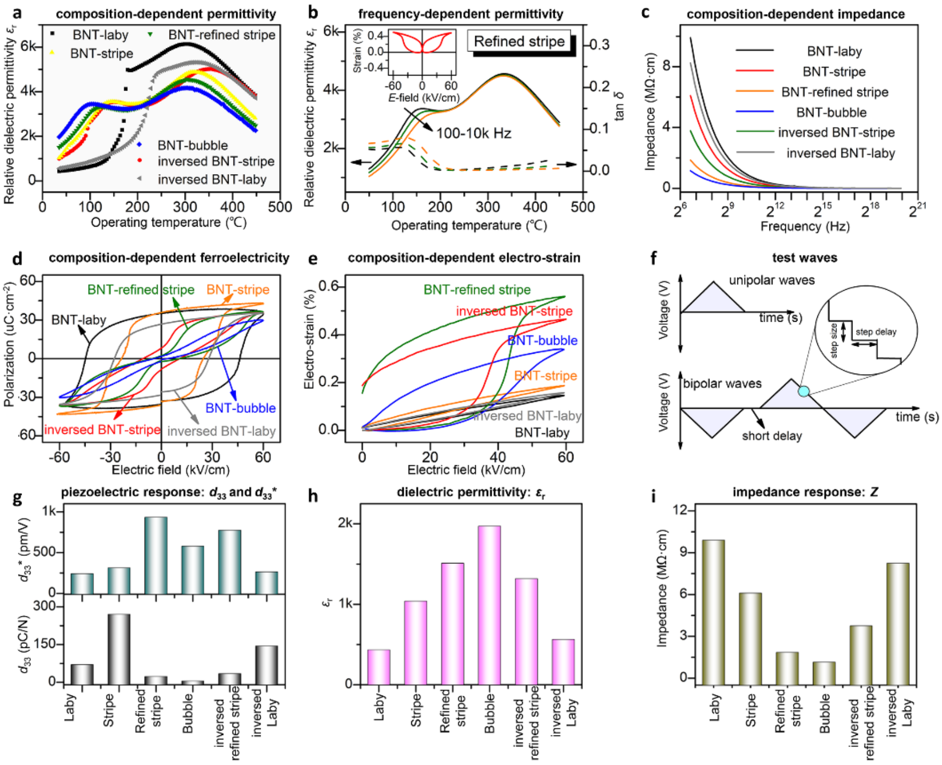

Extended Data Fig. 6 | Reversible electric properties accompanied with the evolutionary domains. **a**, Relative dielectric permittivity (ε_r) against temperatures (25-450 °C, measured at 1k Hz) for BNT-based samples with different domain structures. **b**, Temperature- and frequency-dependent relative dielectric permittivity (ε_r) and dielectric loss (tan δ) for the representative composition sharing the refined stripe domain, with insets showing the corresponding bipolar electro-strain versus electric field curves measured at 1 Hz. The broad and frequency dispersive peaks (marked by the black arrow) demonstrate the relaxor nature of the studied samples, and a giant electro-strain (up to 0.56%) was observed for BNT-samples with the refined stripe domain. **c**, Impedance data against frequencies (100 Hz – 1 MHz) for BNT-based samples with different domain structures, measured at room temperature. **d**, Bipolar ferroelectric polarization versus electric field (*P*-*E*) curves for BNT-based samples with different domain structures, measured at room temperature and 1 Hz. **e**, Unipolar electro-strain versus electric field (*S*-*E*) curves for BNT-based samples with different domain structures, measured at room temperature and 1 Hz. **f**, Unipolar and bipolar triangular waves utilized to study the ferroelectric and electro-strain behaviors for BNT-based samples with different domain structures. **g**, Comparison of large-field signal piezoelectric responses derived from electro-strain curves and small-field signal piezoelectric responses derived from d_{33}-meter, **h**) dielectric permittivity and **i**) impedance responses derived from the HP4294 impedance analyzer.

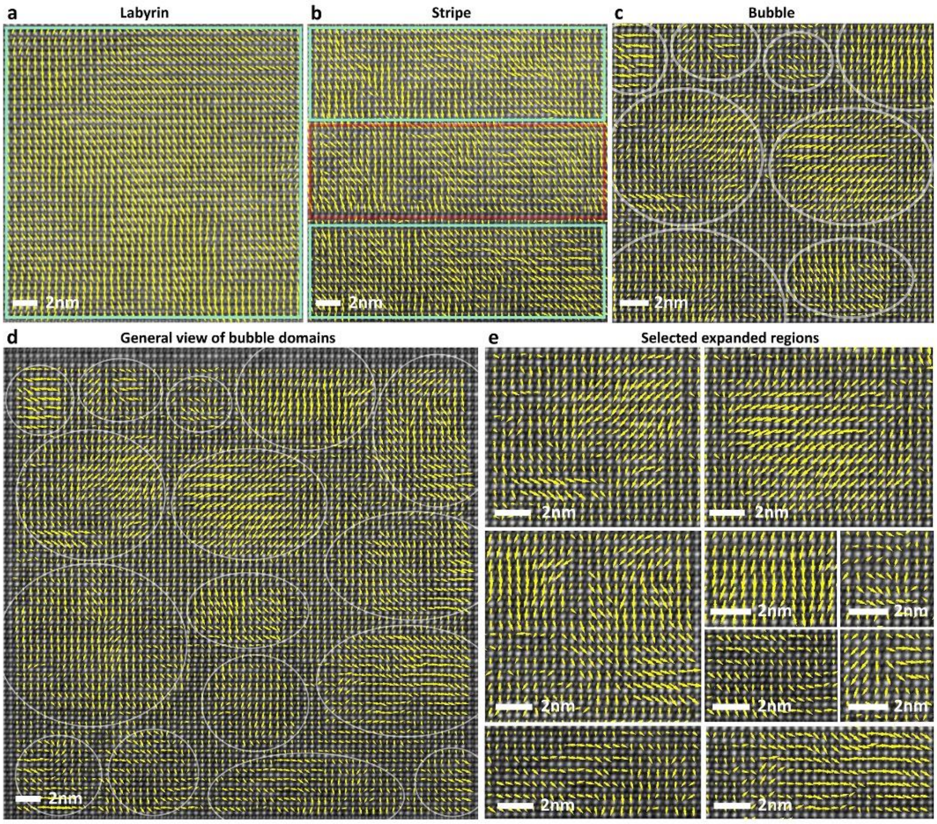

Extended Data Fig. 7 | Experimental observation of the evolutionary domains. a-d, Atomically-resolved STEM ABF (contrast inverted) images of **a**) large-sized BNT-labyrinthine, **b**) BNT-stripe, **c**) BNT-bubble samples and **d**) Bubble domains viewed along $[110]_{pc}$, with the $\delta_{Ti\text{-}O}$ displacement vector maps overlaid on them; the displacement vectors are indicated as yellow arrows. **e**, Enlarged representative regions of the displacement mapping selected in **d**).

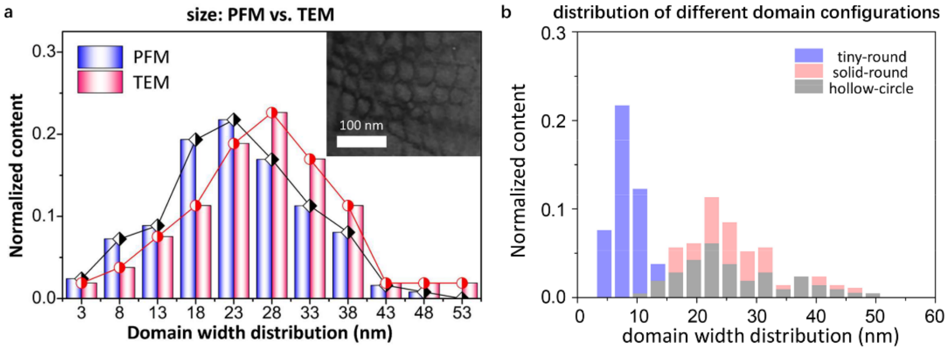

Extended Data Fig. 8 | Width distribution of the bubble domains. (a) Statistics of domain width distribution obtained from PFM and TEM, and the inset showing the bubble domains imaged by TEM. Note here that the data shown in Fig. 8a is derived on the bubble regions with clear boundaries while those without clear boundaries were not adopted. (b) Accumulated statistic of domain width distribution of the three types of domain configurations. Note here that approximate values were utilized to evaluate the width of tiny-round-shaped domains without clear boundaries.

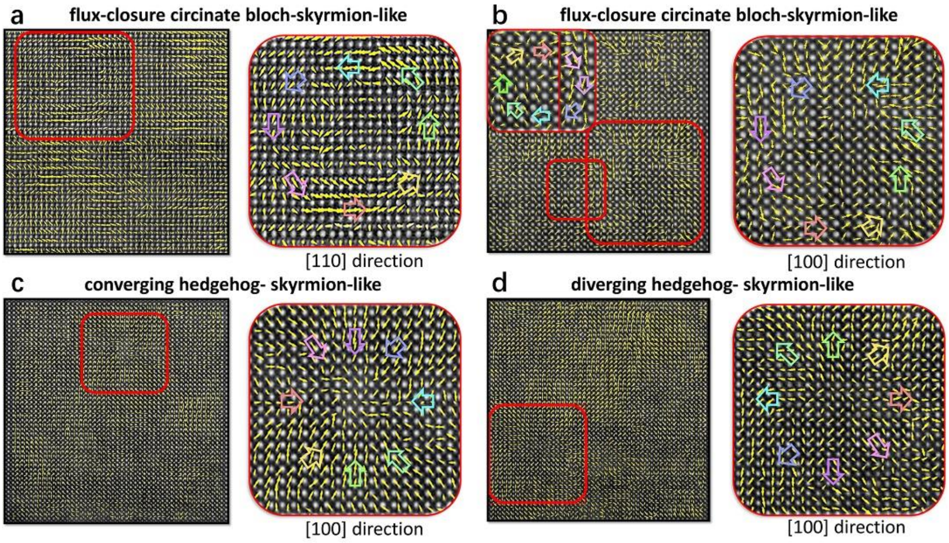

Extended Data Fig. 9 | Skyrmion-like conformations based on the experimentally observed bubble domains. a-d, Atomically-resolved STEM ABF (contrast inverted) images of BNT-bubble samples along $[100]_{pc}$ or $[110]_{pc}$, with the $\delta_{Ti\text{-}O}$ displacement vector maps overlaid on them; the displacement vectors are indicated as yellow arrows; the right images show the enlarged representative area of the displacement mapping selected in the left images. Three representative conformations are selected, including **a**, **b)** the continuous electric dipoles rotation in flux-closure circinate Bloch-skyrmion-like, **c**) converging hedgehog- skyrmion-like and **d**) diverging hedgehog-skyrmion-like topologies.

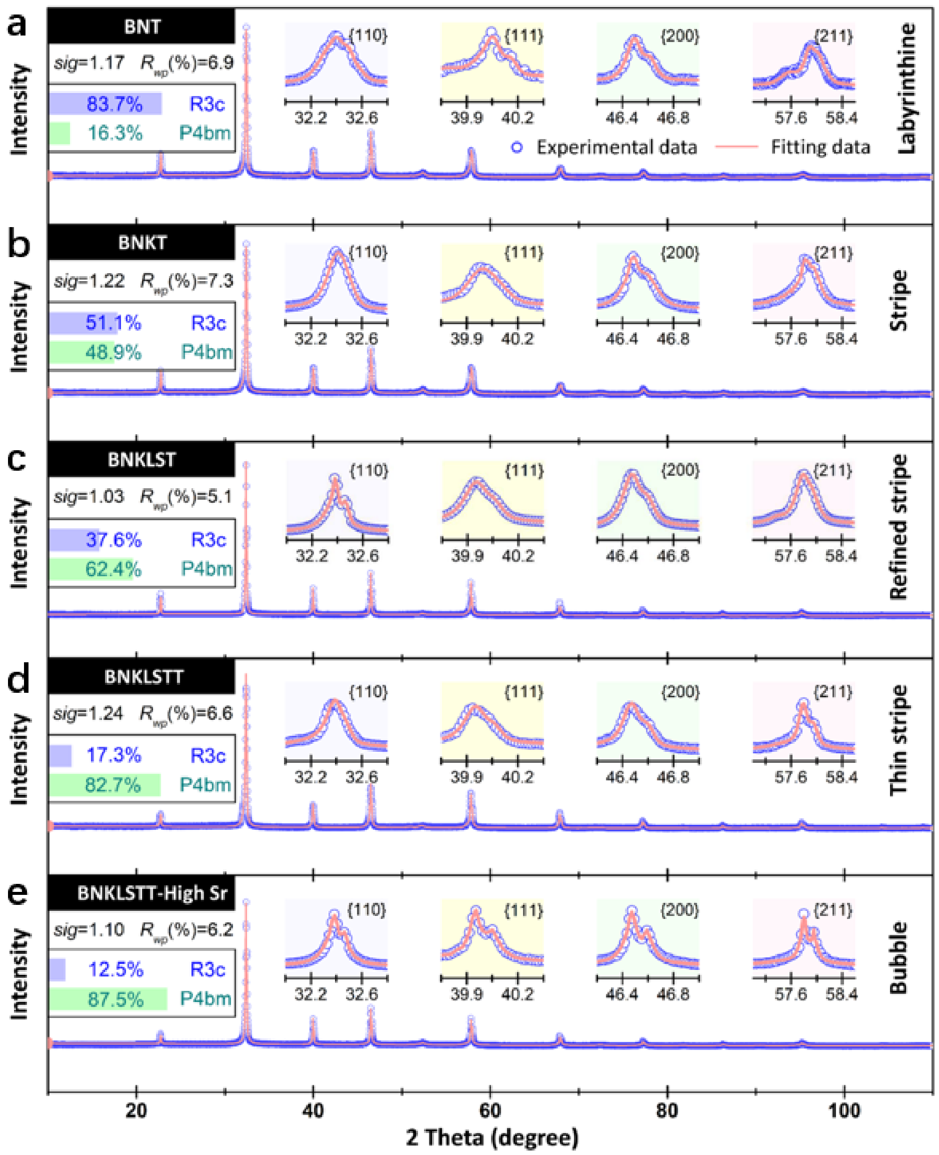

Extended Data Fig. 10 | High-resolution X-ray diffraction Rietveld refinement analysis. P4bm symmetry increases with the increasing doping contents. Insets, expanded characteristic {110}, {111}, {200} and {211} peaks for the corresponding compositions. The refinement results are acceptable when the refinement parameters satisfy the standard Maud requirements (*sig*<2 and R_{wp}<10%).

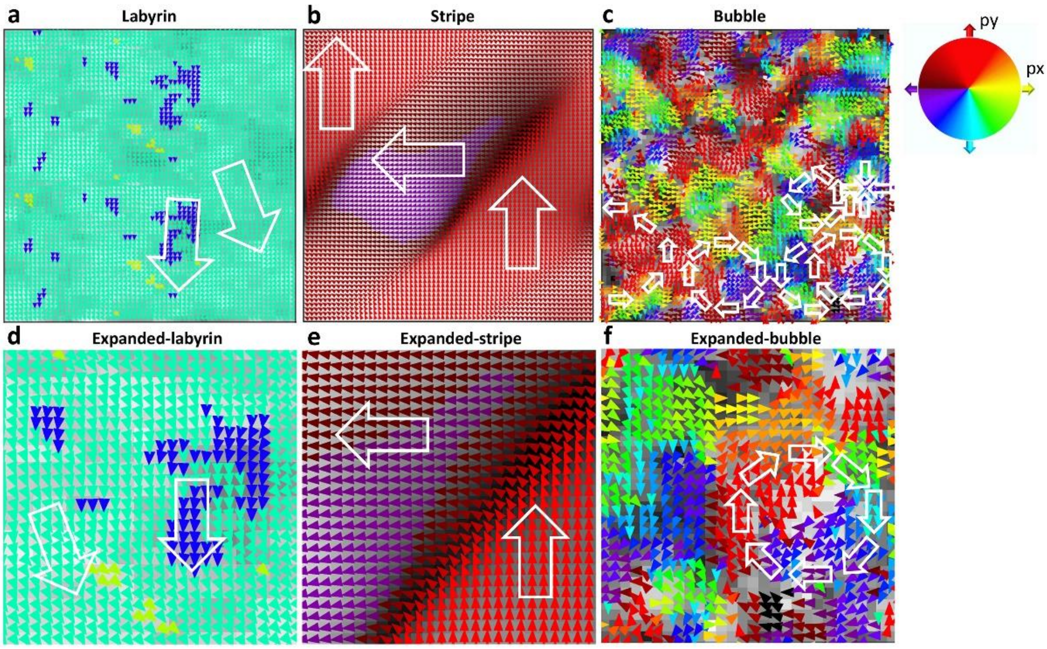

Extended Data Fig. 11 | Theoretical prediction of the evolution of domains. Phase-field modelling results of **a**, large-sized BNT-labyrinthine, **b**, BNT-stripe and **c**, BNT-bubble domains and the corresponding **d-f**, enlarged representative regions of the polarization vectors mapping selected in **a-c**. The polarization vectors are filled with different colors according to their angles (left), which exhibit a continuous distribution (right). The grid background shows the corresponding magnitude of each vector.

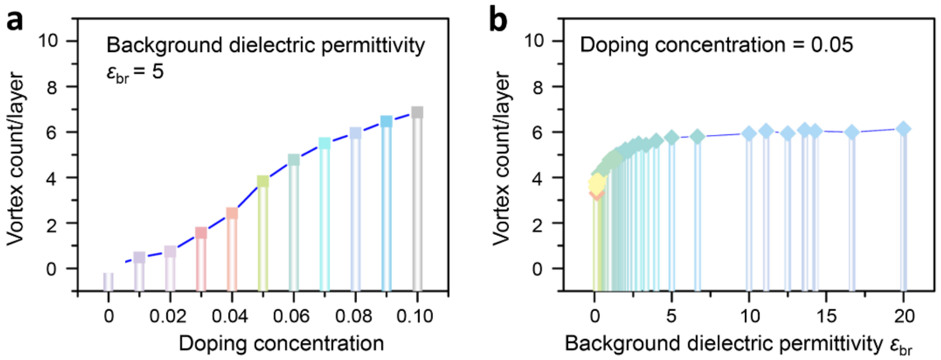

Extended Data Fig. 12 | Main driving forces of the bubble domains. The densities of vortices as the function of **a**, doping concentration and **b**, background dielectric permittivity ε_{br}. When we study the effect of one parameter, other parameters are kept constant.

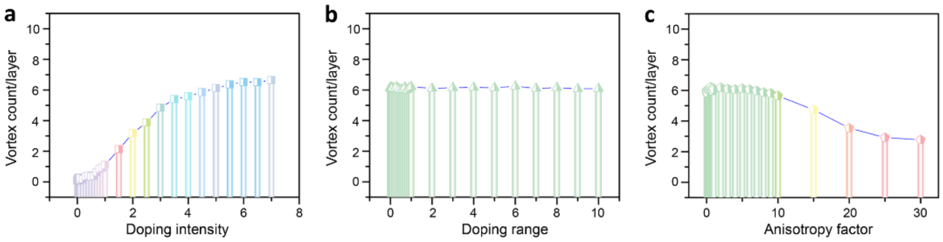

Extended Data Fig. 13 | Driving forces of the bubble domains. The densities of vortexes as the function of **a**, doping intensity, **b**, doing range, **c**, anisotropy factor. When we study the effect of one parameter, other parameters are kept constant.

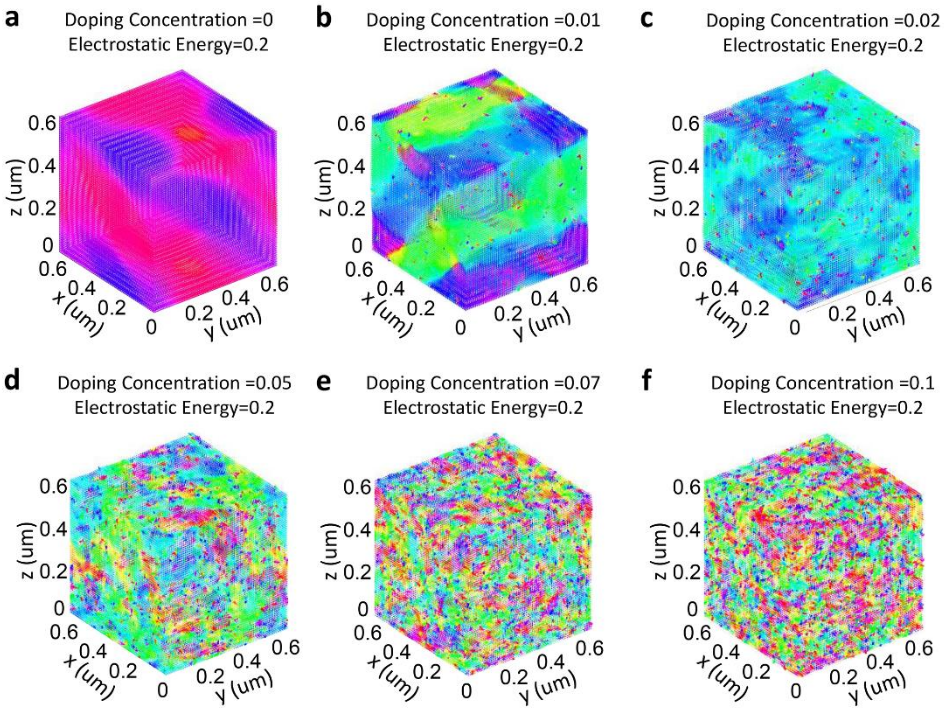

Extended Data Fig. 14 | Effect of doping concentration on domain evolution. a-f, The domain morphology evolves with increasing doping concentration from 0 to 0.1. When we study the effect of one parameter, other parameters are kept constant.

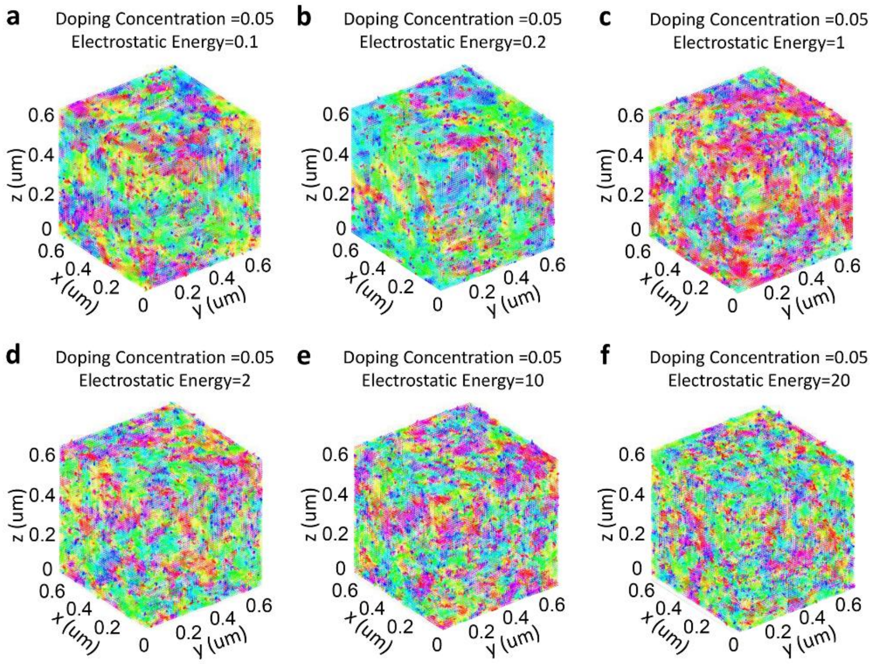

Extended Data Fig. 15 | Effect of electrostatic parameter ε_{br} on domain evolution. a-f, The domain morphology evolves with increasing electrostatic parameter ε_{br} from 0.1 to 20. When we study the effect of one parameter, other parameters are kept constant.

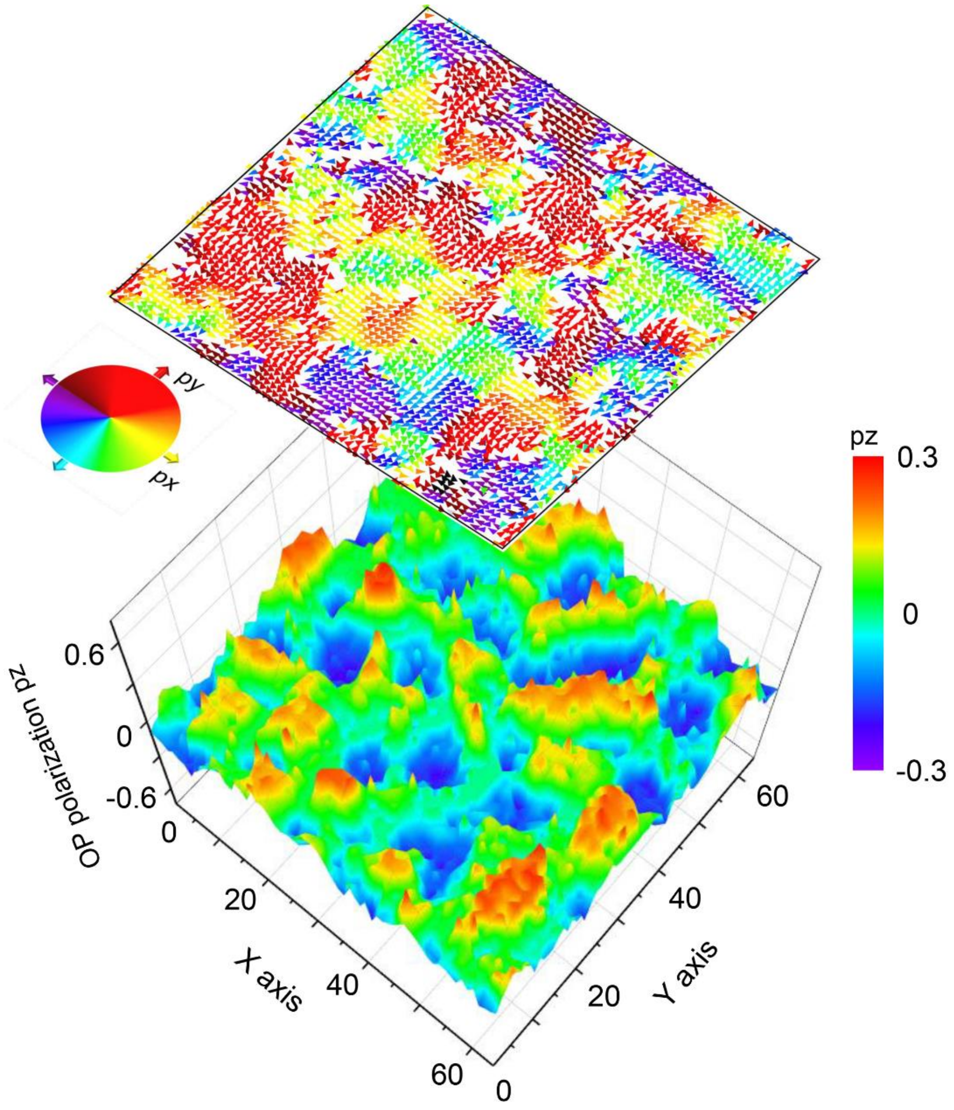

Extended Data Fig. 16 | Theoretical reconstruction of bubble domains. Phase-field modelling results to reconstruct the 3D structure of the experimentally observed bubble domains. The upper 2D picture shows the polarization vector distribution projected onto the XY plane, and the lower 3D picture shows the 3D structure of the bubble domains viewed from the out-of-plane direction (consistent with the experimental observations).

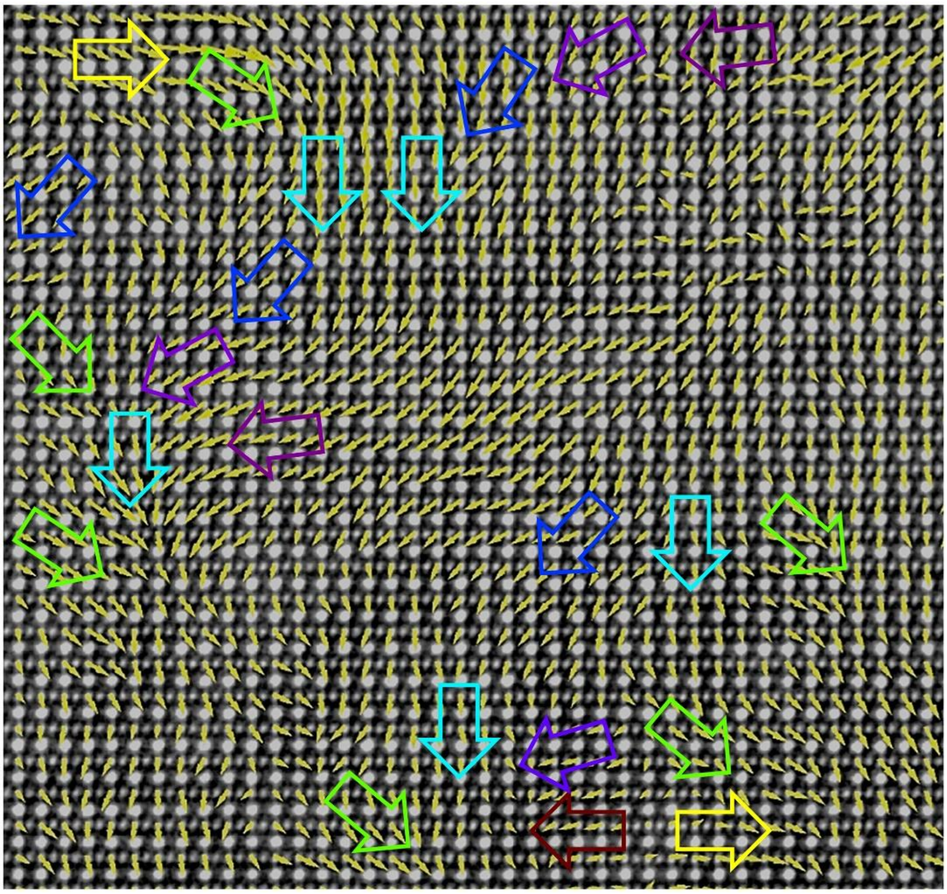

Extended Data Fig. 17| Other conformations of the experimentally observed bubble domains. Atomically-resolved STEM ABF (contrast inverted) images of BNT-bubble samples along $[100]_{pc}$, with the $\delta_{Ti\text{-}O}$ displacement vector mapping overlaid on them; the displacement vectors are indicated as yellow arrows. The colored arrows indicate the elusive polarization distribution in local regions.

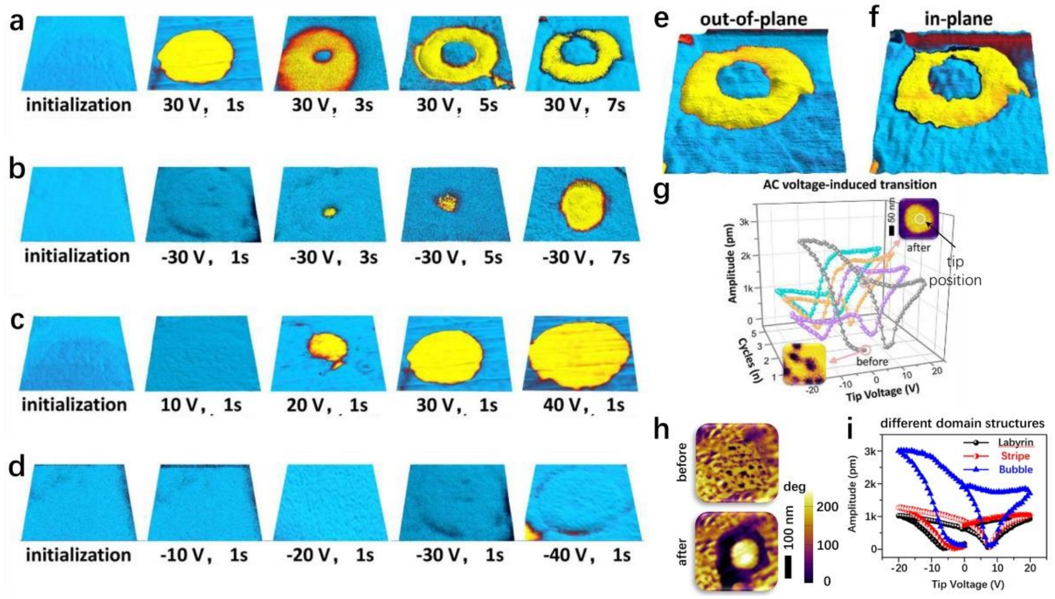

Extended Data Fig. 18 | D.C. and A.C. voltage-induced evolution from the bubble domains. a-d, Initial poling is achieved by scanning a 1*1 μm^2 area with –40 V applied to the tip. Applying D.C. voltages to the center points for different times then induces the domain morphologies shown: **a**) (+30 V, 1 to 7 s), **b**) (10 to 40 V, 1 s), **c**) (–30 V, 1 to 7 s) and **d**) (–10 to –40 V, 1 s). **e**, Out-of-plane and **f**, in-plane domain morphology of the donut-like domain morphology. **g**, Local piezoelectric responses derived from switching spectroscopic PFM (SSPFM) hysteresis loops performed on bubble domains. Insets show the A.C. voltage-induced irreversible transition from the bubble state to the cylindrical state (phase signal). **h**, Larger scale PFM images show the A.C. voltage-induced irreversible transition from the bubble state to the cylindrical state (phase signal). Tests are carried out under dry atmosphere condition (humidity=0%). **i**, Local piezoelectric responses derived from switching spectroscopic PFM (SSPFM) hysteresis loops performed on labyrinthine, stripe and bubble domains structures.

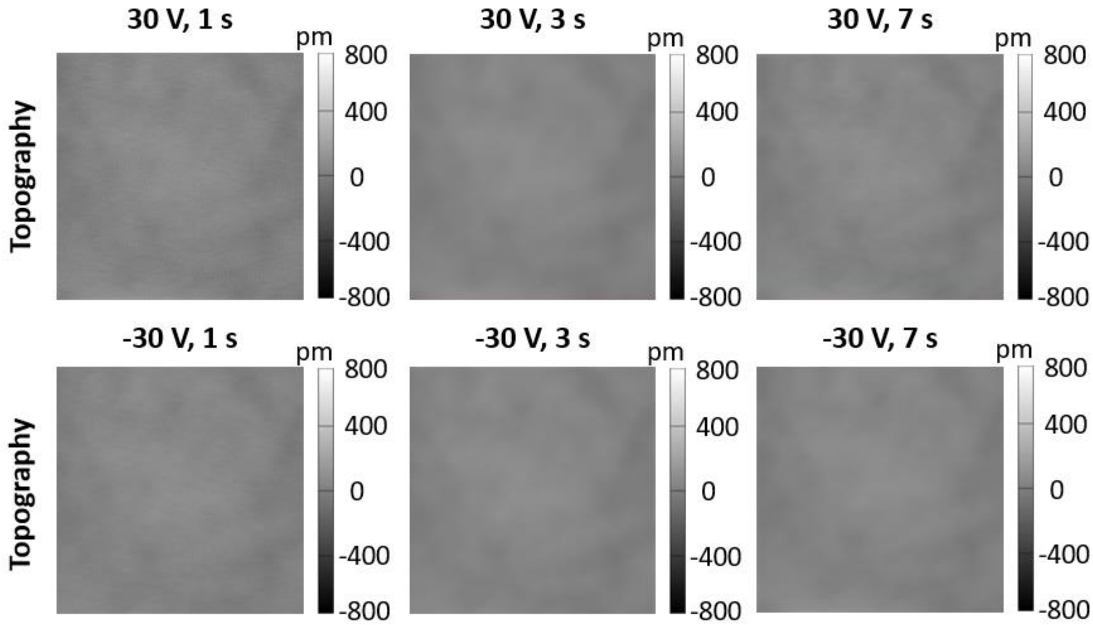

Extended Data Fig. 19 | The topographic images with different duration time of tip voltage.

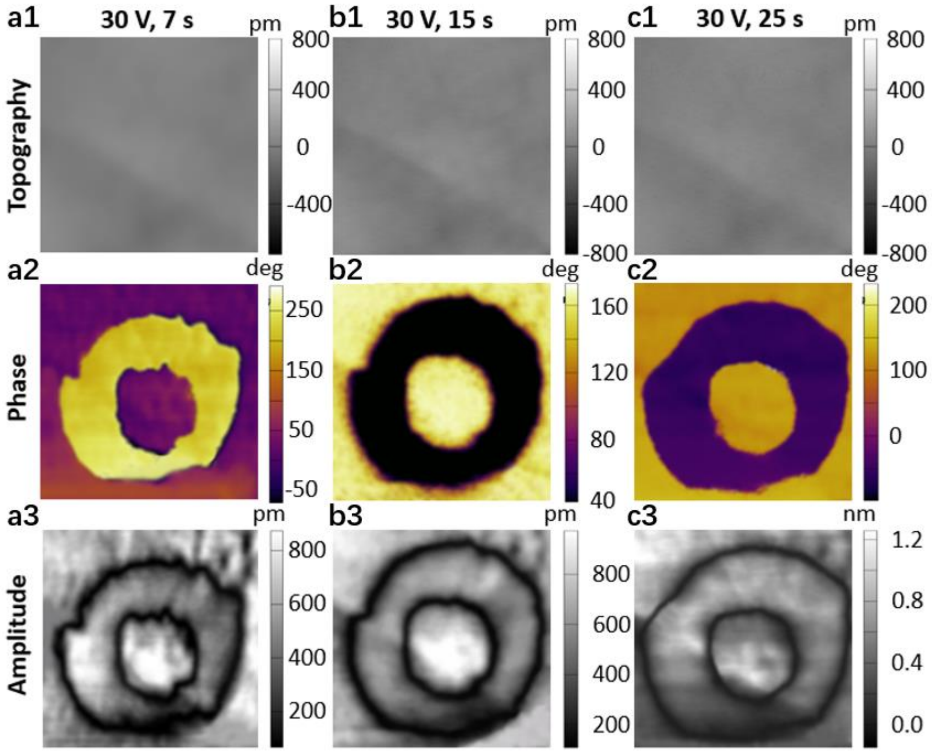

Extended Data Fig. 20 | a1-c1, Topography, **a2-c2,** phase and **a3-c3,** amplitude images with the increasing duration time.

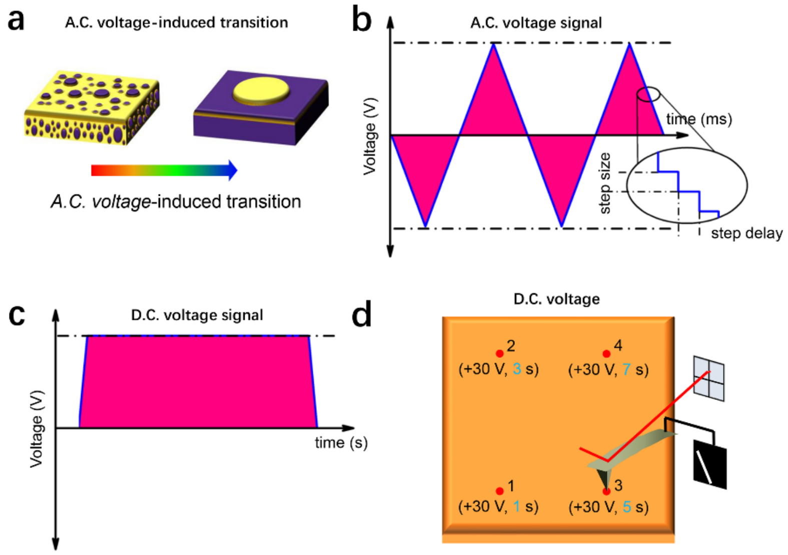

Extended Data Fig. 21 | Test waves for A.C. and D.C. voltage-dependent PFM. a, Schematic illustration of the A.C. voltage-induced transition from the bubble state to the cylindrical state. Test waves of **b**, triangular alternating voltage and **c**, D.C. voltage utilized for the local SSPFM test. **d**, Schematic illustration showing how to apply the tip voltage on the samples.

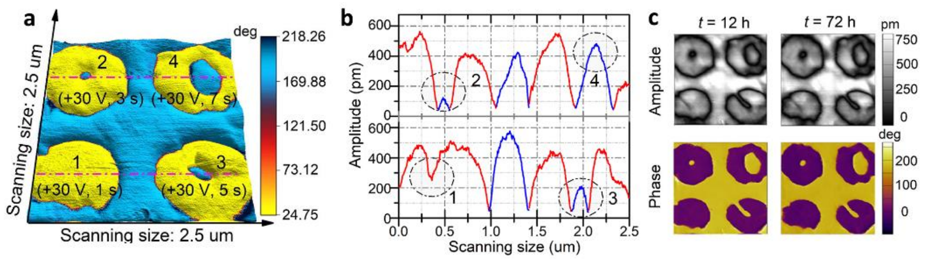

Extended Data Fig. 22 | D.C. voltage-induced donut-like domains on the initial bubble domains. a, Donut-like domain morphology obtained after a tip D.C. voltage of (+30 V, 1 to 7 s) was applied on the selected points. Before the tip voltage was applied to the selected points, (–50 V, 1s) was applied over an area of 3*3 μm^2. **b**, Detailed piezo-response data derived from the images of 16a. Regions marked by circles indicate anti-parallel polarization directions. **c**, Spatial stability of the voltage-induced donut-like domains. After aging treatments of 12 hours and 72 hours, these donut-like ferroelectric domains are still stable. Tests are carried out under dry atmosphere condition (humidity=0%).

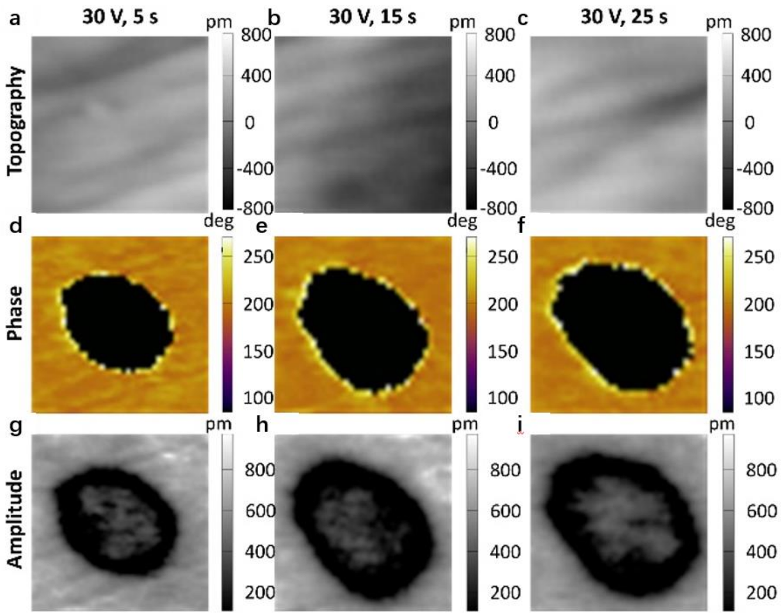

Extended Data Fig. 23 | Domain morphology of BNT-stripe domain induced by the tip voltage. There are no doughnut-like domains formed like that in the BNT-bubble samples.

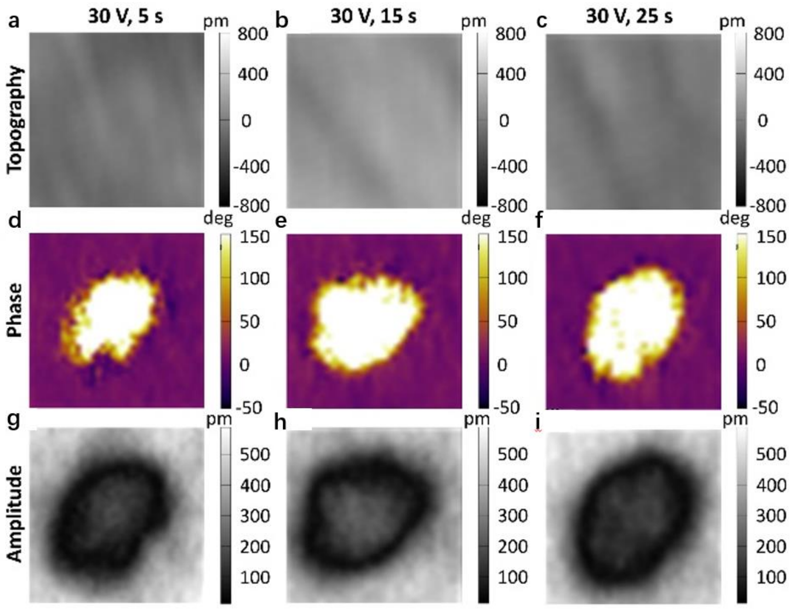

Extended Data Fig. 24 | Domain morphology of BNT-labyrinthine domain induced by the tip voltage. There are no doughnut-like domains formed like that in the BNT-bubble samples.

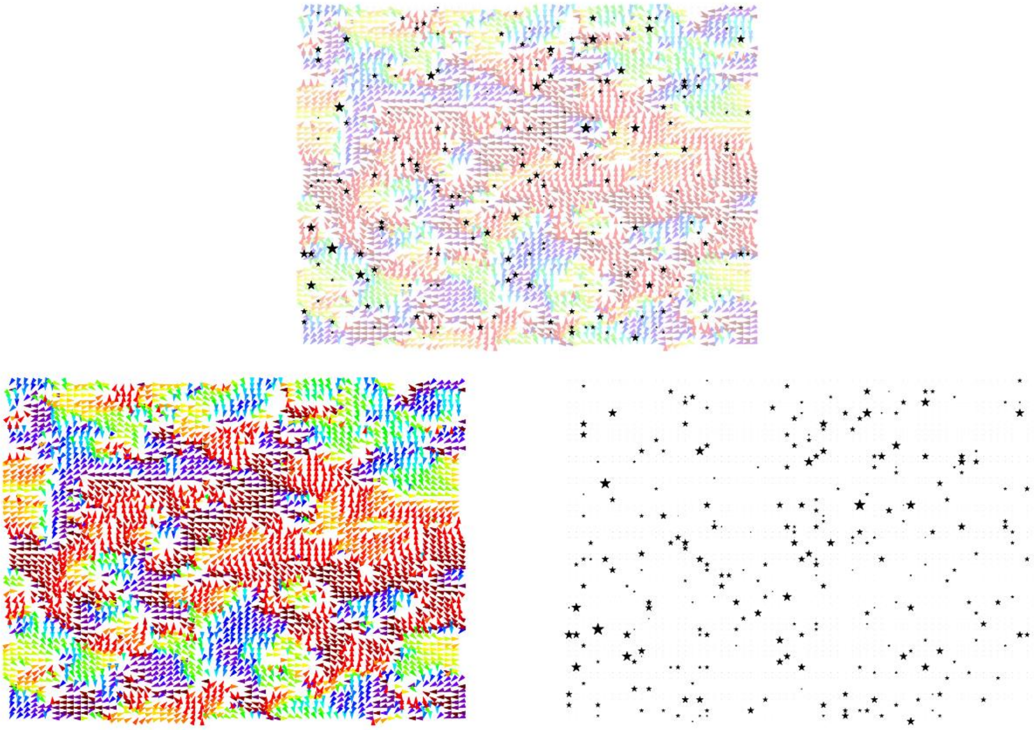

Extended Data Fig. 25 | The effects of defects. Spatially distribution of doping defects and polar vectors from phase field simulations. The black stars label the location of doping defects while the size of the stars presents the intensity of defects and the colored arrows show the polar vectors in each mesh point.

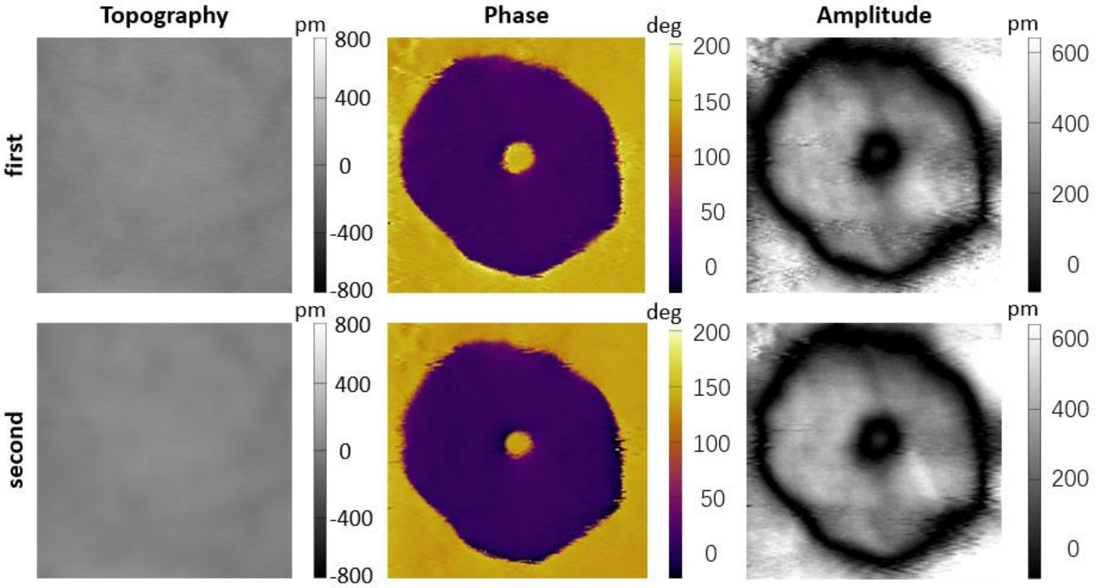

Extended Data Fig. 26 | The reversibility of tip voltage-induced domain morphologies. The voltage-induced domain evolution on BNT bubble domains is reversible.

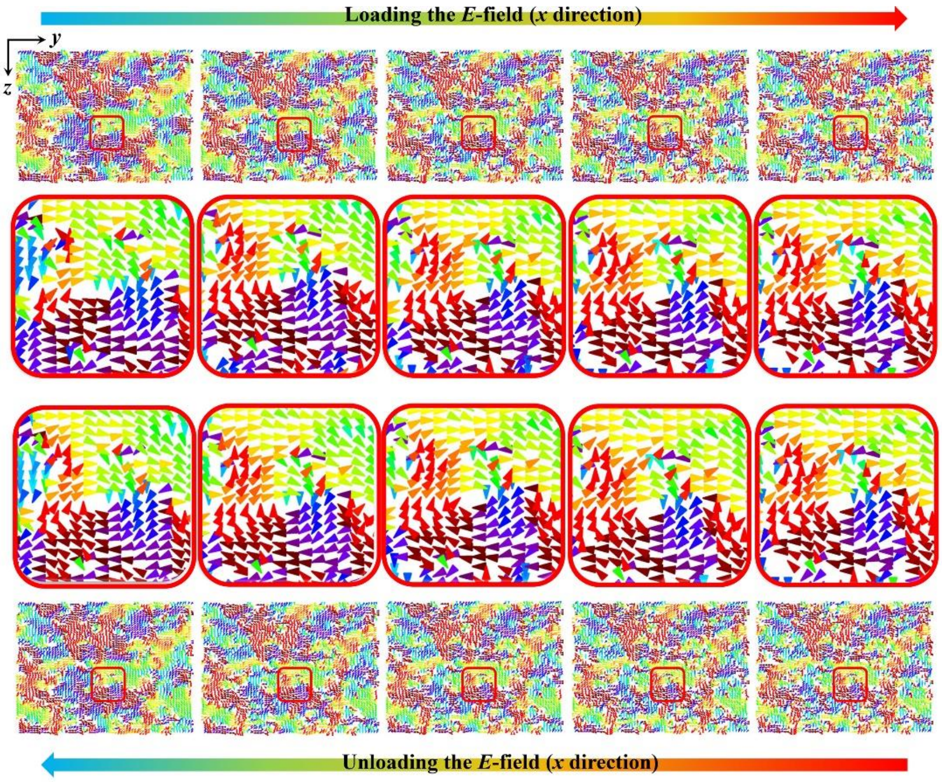

Extended Data Fig. 27 | Domain evolution under external electric field. Phase field simulation of domain-pattern evolution (*yz* plane) upon an electric field (*x* direction) loading-unloading cycle. The red box labels the selected skyrmion-like polar structures which are expanded to a larger scale for a better view.

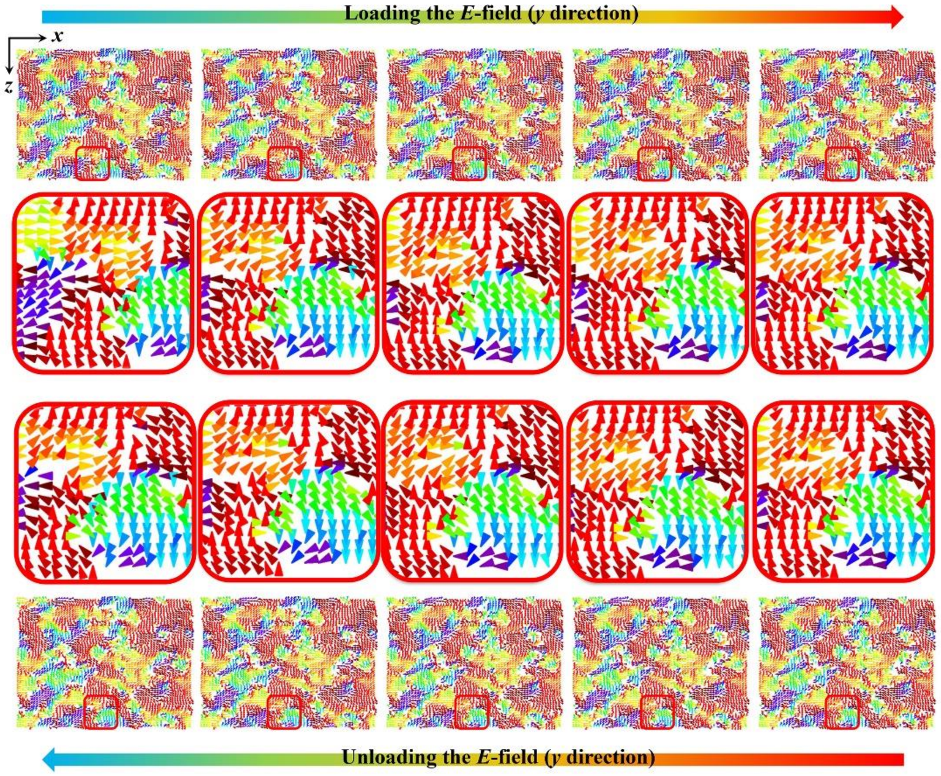

Extended Data Fig. 28 | Domain evolution under external electric field. Phase field simulation of domain-pattern evolution (*xz* plane) upon an electric field (*y* direction) loading-unloading cycle. The red box labels the selected skyrmion-like polar structures which are expanded to a larger scale for a better view.

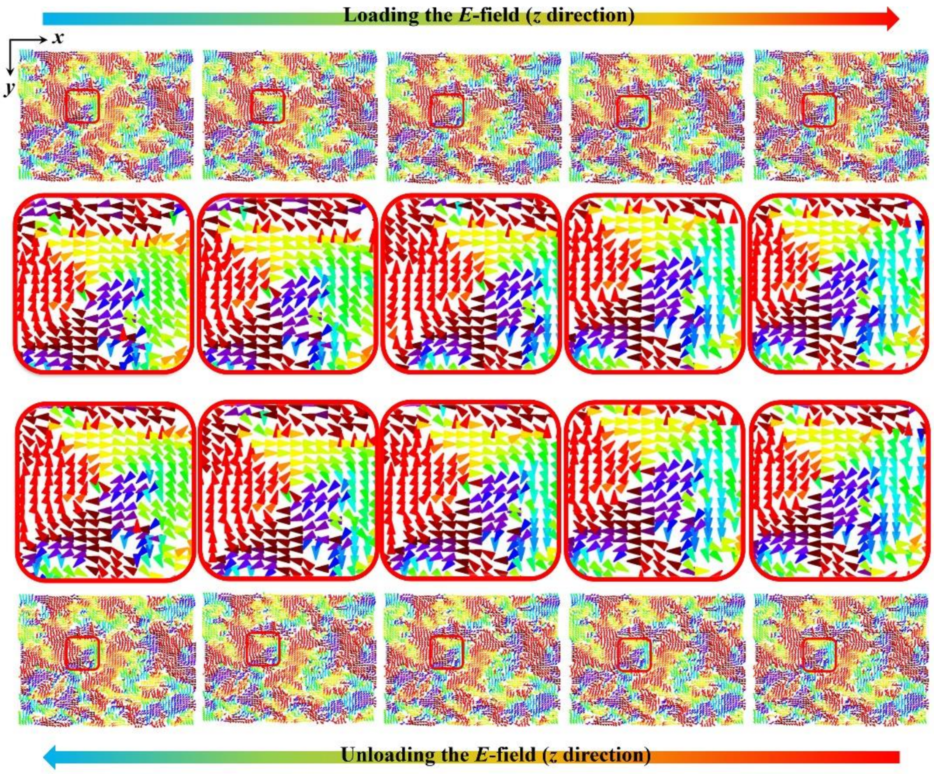

Extended Data Fig. 29 | Domain evolution under external electric field. Phase field simulation of domain-pattern evolution (*xy* plane) upon an electric field (*z* direction) loading-unloading cycle. The red box labels the selected skyrmion-like polar structures which are expanded to a larger scale for a better view.

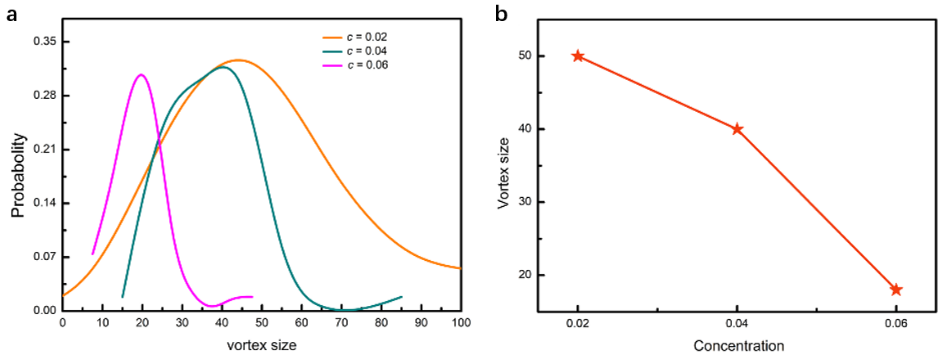

Extended Data Fig. 30 | The effects of doping concentration on the size and density of bubble domains. Dependence of the size of vortex structures on doping concentration. (a) The probability distribution of the size of vortex structures; (b) the mean size of vortex structures as a function of doping concentration.

Extended Data Table 1. Density of studied $Bi_{0.5}Na_{0.5}TiO_3$-based ceramics. The measured density is in an ascending sort order.

	BNT-labyrin	BNT-stripe	BNT-refined stripe	BNT-bubble	inversed BNT-refined stripe	inversed BNT-labyrin
Measured density (g cm^{-3})	5.7247	5.7323	5.6278	5.5171	5.5547	5.6071
	5.7713	5.7747	5.7091	5.6368	5.6672	5.6997
	5.7911	5.7931	5.7564	5.7619	5.7348	5.7574
	5.8117	5.8203	5.8079	5.7803	5.7921	5.8107
	5.8324	5.8429	5.8181	5.8101	5.8238	5.8337
Average (g cm^{-3})	5.7862	5.7927	5.7439	5.7012	5.7145	5.7417
Error (%)	1.86	1.91	3.31	5.14	4.71	3.95

Extended Data Table 2. Material coefficients of $Bi_{0.5}Na_{0.5}TiO_3$-based materials utilized for the phase-field modelling in this work.

Parameter	Value	Unit
α_1	$(T\text{-}381)3.34\times10^5$	$Jm.C^{-2}$
α_{11}	$(T\text{-}393)4.69\times10^6\text{-}2.02\times10^8$	$Jm^5.C^{-4}$
α_{12}	3.23×10^8	$Jm^5.C^{-4}$
α_{111}	$(393\text{-}T)5.52\times10^7+2.76\times10^9$	$Jm^9.C^{-6}$
α_{112}	4.47×10^9	$Jm^9.C^{-6}$
α_{123}	4.91×10^9	$Jm^9.C^{-6}$
c_{11}	27.5×10^{10}	Jm^{-3}
c_{12}	17.9×10^{10}	Jm^{-3}
c_{44}	5.43×10^{10}	Jm^{-3}
Q_{11}	0.1104	$m^4.C^{-2}$
Q_{12}	-0.0452	$m^4.C^{-2}$
Q_{44}	0.0578	$m^4.C^{-2}$
G_{11}	51×10^{-11}	$Jm^3.C^{-2}$
G_{12}	-2×10^{-11}	$Jm^3.C^{-2}$
G_{44}	2×10^{-11}	$Jm^3.C^{-2}$
Γ	4×10^4	$C^2J^{-1}m^{-1}s^{-1}$

References

33. Kwei, G. H., Lawson, A. C., Billinge, S. J. L. & Cheong, S. W. Structures of the ferroelectric phases of barium titanate. *J. Phys. Chem.* **97**, 2368-2377 (1993).

34. Harada, J., Pedersen, T. & Barnea, Z. X-ray and neutron diffraction study of tetragonal barium titanate. *Acta Crystallogr. A* **26**, 336-344, (1970).

35. Jia, C.-L., Urban, K. W., Alexe, M., Hesse, D. & Vrejoiu, I. Direct Observation of Continuous Electric Dipole Rotation in Flux-Closure Domains in Ferroelectric Pb(Zr,Ti)O_3. *Science* **331**, 1420 (2011).

36. Jia, C.-L. *et al.* Unit-cell scale mapping of ferroelectricity and tetragonality in epitaxial ultrathin ferroelectric films. *Nat. Mater.* **6**, 64-69, (2007).

37. Jia, C. L. *et al.* Effect of a Single Dislocation in a Heterostructure Layer on the Local Polarization of a Ferroelectric Layer. *Phys. Rev. Lett.* **102**, 117601 (2009).

38. Wei, X.-K. *et al.* Néel-like domain walls in ferroelectric Pb(Zr, Ti)O_3 single crystals. *Nat. Commun.* **7**, 12385, (2016).

39. Peters, J. J. P., Apachitei, G., Beanland, R., Alexe, M. & Sanchez, A. M. Polarization curling and flux closures in multiferroic tunnel junctions. *Nat. Commun.* **7**, 13484, (2016).

40. Yang, Y. *et al.* Doping effects of point defects in shape memory alloys. *Acta Mater.* **176**, 177-188 (2019).

41. Sluka, T., Tagantsev, A. K., Damjanovic, D., Gureev, M. & Setter, N. Enhanced electromechanical response of ferroelectrics due to charged domain walls. *Nat .Commun.* **3**, 1-7 (2012).

42. Berg, B. & Lüscher, M. Definition and statistical distributions of a topological number in the lattice O (3) σ-model. *Nucl. Phys. B* **190**, 412-424 (1981).

43. Shvartsman, V., Dkhil, B. & Kholkin, A. Mesoscale domains and nature of the relaxor state by piezoresponse force microscopy. *Annu. Rev. Mater. Res.* **43**, 423-449 (2013).

44. Gregg, J. M. Exotic Domain States in Ferroelectrics: Searching for Vortices and Skyrmions. *Ferroelectrics* **433**, 74-87, (2012).

45. Zhang, Q. *et al.* Nanoscale Bubble Domains and Topological Transitions in Ultrathin Ferroelectric Films. *Adv. Mater.* **29**, (2017).

46. Pirc, R. & Blinc, R. Spherical random-bond–random-field model of relaxor ferroelectrics. *Phys. Rev. B* **60**, 13470 (1999).

47. Glazounov, A., Tagantsev, A. & Bell, A. Evidence for domain-type dynamics in the ergodic phase of the $PbMg_{1/3}Nb_{2/3}O_3$ relaxor ferroelectric. *Phys. Rev. B* **53**, 11281 (1996).

48. Westphal, V., Kleemann, W. & Glinchuk, M. Diffuse phase transitions and random-field-induced domain states of the "relaxor"ferroelectric $PbMg_{1/3}Nb_{2/3}O_3$. *Phys. Rev. Lett.* **68**, 847 (1992).

49. Vugmeister, B. & Rabitz, H. Coexistence of the critical slowing down and glassy freezing in relaxor ferroelectrics. *Phys. Rev. B* **61**, 14448 (2000).

50. Vugmeister, B. E. & Glinchuk, M. D. Dipole glass and ferroelectricity in random-site electric dipole systems. *Rev. Mod. Phys.* **62**, 993-1026 (1990).

51. Jaffe, B., Cook, W. & Jaffe, H. Piezoelectric Ceramics (Academic Press, London, 1971).

52. Kézsmárki, I. *et al.* Néel-type skyrmion lattice with confined orientation in the polar magnetic semiconductor GaV_4S_8. *Nat. Mater.* **14**, 1116-1122 (2015).

53. Taranenko, V. B., Staliunas, K., & Weiss, C. O. Pattern formation and localized structures in degenerate optical parametric mixing. *Phys. Rev. Lett.* **81**, 2236 (1998).

54. Qiao, H. *et al.*. Orientation-dependent electrical property and domain configuration of Mn-doped

$Pb(In_{0.5}Nb_{0.5})O_3$–$PbTiO_3$ single crystal. *J. Am. Ceram. Soc.* **102**, 79-84 (2019).
